# Supplementary material for: Chemometric Models of Differential Amino Acids at the Navα and Navβ Interface of Mammalian Sodium Channel Isoforms
Source: Molecules. 2020 Aug 3;25(15):3551. doi: 10.3390/molecules25153551 (PMC7435598; doi:10.3390/molecules25153551)
Supplement: Supplementary file 1 [file molecules-25-03551-s001.pdf]

molecules

MDPI

Supplementary Materials

Chemometric Models of Differential Amino Acids at the Na$_v$α and Na$_v$β Interface of Mammalian Sodium Channel Isoforms

Fernando Villa-Diaz [1], Susana Lopez-Nunez [1], Jordan E. Ruiz-Castelan [1], Eduardo Marcos Salinas-Stefanon [2] and Thomas Scior [1,*]

[1] Laboratory of Computational Molecular Simulations, Faculty of Chemical Sciences, BUAP, C.P. 72570 Puebla, Pue., Mexico; fer.vdl1928@gmail.com (F.V.-D.); susannalopez18@gmail.com (S.L.-N.); toj_cdai90@live.jp (J.E.R.-C.)

[2] Institute of Physiology, BUAP, C.P. 72570 Puebla, Pue., Puebla, Mexico; eduardo.salinas@correo.buap.mx

* Correspondence: tscior@gmail.com; thomas.scior@correo.buap.mx; Tel.: 52 -222-2 295500 ext. 7529

Supplementary Materials

The Supplementary Material (SM) was necessary to document the essential findings as a synopsis taken from an even larger number of systematically conducted chemometric studies on the Sodium channels:

1. **Multiple Sequence Alignments (MSA): 9 (isoforms) x 3 (species) x 16 (ECLs) = 432 primary sequences.**
2. **3D models of 3D templates and homology models: 9 x 3 x 2 (β1,β3) = 54 PDB files.**
3. **Calculations of surfaces: 9 x 3 x 16 = 432 surface models.**
4. **Calculations of polar and non-polar surfaces: (9 x 3 x 16) x 2 (PSA, nonPSA) = 432 x 2 properties.**
5. **Calculations of molecular volumes: 9 x 3 x 16 = 432 volume patterns.**
6. **Interface area calculation (buried area): 9 x 3 x 2 (α / β) x 4 (ECLs)= 216 numbers of buried regions between subunits α and β.**
7. **Calculations of IF area of nine isoforms by three species by four IF-ECLs: 9 x 3 x 2 (β1,β3) x 4 (IF-ECLs) x 2 (α / β) = 432 total number of surface patterns.**
8. **Calculations of IF area concerning the polar and nonpolar atoms that form the interface: 9 x 3 x 2 (β1,β3) x 4 (IF-ECLs) x 2 (α / β) = 432 polar and 432 nonpolar atomic patterns.**
9. **Calculations of the MEPS area (red = negative, blue = positive) based on the vertices of the solvent-accessible surface of the atoms that make up the interface: 9 x 3 x 2 (β1,β3) x 4 (IF-ECLs) x 2 (α / β) = 432 positive charge patterns and 432 negative charge patterns.**
10. **Counting of the loop lengths: 9 (isoforms) x 3 (species) x 16 (ECLs) = 432 counts.**
11. **Counting of the polar residues: 9 (isoforms) x 3 (species) x 16 (ECLs) = 432 counts.**
12. **Counting of the cysteine residues as potential formation of S-S bridges: 9 (isoforms) x 3 (species) x 16 (ECLs) = 432 counts.**
13. **Counting of the aromatic, positively or negatively charged residues: 9 (isoforms) x 3 (species) x 16 (ECLs) x 3 = 432 x 3 counts.**

Table S1. PPI of the residues of the Na$_v$α and Na$_v$β subunits

Isoform	ECL	S5 DI		S1-S2 DIII		S5 DIV	S6 DIV		
	PPI Id[3]	1	2	3	4	5	6	7	8
hNa$_v$1.1[7]	P35498[2]	agqCpeg(O)	qcpEgym(O)	yidQrkt(N)	dqrKtik(N)	gidDmfn(O)	PnkVnpg[NI]	nkvNpgs(O)	kvnPgss(O)
mNa$_v$1.1[7]	A2APX8[2]	agqCpeg(O)	qcpEgym(O)	yidQrkt(N)	dqrKtik(N)	gidDmfn(O)	pnkVnpg[NI]	nkvNpgs(O)	kvnPgss(O)
rNa$_v$1.1[7]	P04774[2]	agqCpeg(O)	qcpEgym(O)	yidQrkt(N)	dqrKtik(N)	gidDmfn(O)	pnkVnpg[NI]	nkvNpgs(O)	kvnPgss(O)
hNa$_v$1.2[5]	6J8E[1]	agqCpeg(O)	qcpEgyi(O)	yieQrkt(N)	eqrKtik(N)	gidDmfn(O)	pdkDhpg(O)	dkdHpgs(N)	kdhPgss(O)
mNa$_v$1.2[7]	B1AWN6	agqCpeg(O)	qcpEgyi(O)	yieQrkt(N)	eqrKtik(N)	gidDmfn(O)	pekDhpg(O)	ekdHpgs(N)	kdhPgss(O)
rNa$_v$1.2[7]	P04775[2]	agqCpeg(O)	qcpEgyi(O)	yieQrkt(N)	eqrKtik(N)	gidDmfn(O)	pekDhpg(O)	ekdHpgs(N)	kdhPgss(O)
hNa$_v$1.3[7]	Q9NY46[2]	agqCpeg(O)	qcpEgyi(O)	yieQrkt(N)	eqrKtik(N)	gidDmfn(O)	pdtIhpg[NI]	dtiHpgs(N)	tihPgss(O)
mNa$_v$1.3[7]	A2ASI5[2]	agqCpeg(O)	qcpEgyi(O)	yieQrkt(N)	eqrKtik(N)	gidDmfn(O)	pdaIhpg[NI]	daiHpgs(N)	aihPgss(O)
rNa$_v$1.3[7]	P08104[2]	agqCpeg(O)	qcpEgyi(O)	yieQrkt(N)	eqrKtik(N)	gidDmfn(O)	pdaIhpg[NI]	daiHpgs(N)	aihPgss(O)
eeNa$_v$1.4[5]	5XSY[1]	agkCpeg[NI]	kcpEgyt(O)	yiwRrrv(N)	wrrRvik(N)	gvdDifn(O)	pdvEnpg(O)	dveNpgt(O)	venPgtd(O)
hNa$_v$1.4[5]	6AGF[1]	aghCpeg(O)	hcpEgye(O)	yieQrrv(N)	eqrRvir(N)	gidDmfn(O)	pnlEnpg(O)	nleNpgt(O)	lenPgts(O)
mNa$_v$1.4[7]	Q9ER60[2]	aghCpeg(O)	hcpEgye(O)	yieQrrv(N)	eqrRvir(N)	gidDmfn(O)	ptlEnpg(O)	tleNpgt(O)	lenPgtn(O)
rNa$_v$1.4[7]	P15390[2]	aghCpeg(O)	hcpEgye(O)	yieQrrv(N)	eqrRvir(N)	gidDmfn(O)	ptlEnpg(O)	tleNpgt(O)	lenPgtn(O)
hNa$_v$1.5[7]	Q14524[2]	agtCpeg(O)	tcpEgyr(O)	yleErkt[NI]	eerKtik(N)	gidDmfn(O)	ptlPnsn[NI]	tlpNsng(O)	lpnSngs(O)
mNa$_v$1.5[7]	Q9JJV9[2]	agtCpeg(O)	tcpEgyr(O)	yleErkt[NI]	eerKtik(N)	gidDmfn(O)	pnlPnsn[NI]	nlpNsng(O)	lpnSngs(O)
rNa$_v$1.5[5]	6U70[1]	agtCpeg(O)	tcpEgyr(O)	yleErkt[NI]	eerKtik(N)	gidDmfn(O)	pnlPnsn[NI]	nlpNsng(O)	lpnSngs(O)
hNa$_v$1.6[7]	Q9UQD0	agqCpeg(O)	qcpEgyq(O)	yieQrkt(N)	eqrKtir(N)	gidDmfn(O)	ldkEhpg(O)	dkeHpgs (N)	kehPgsg(O)
mNa$_v$1.6[7]	Q9WTU3	agqCpeg(O)	qcpEgfq(O)	yieQrkt(N)	eqrKtir(N)	gidDmfn(O)	ldkEhpg(O)	dkeHpgs(N)	kehPgsg(O)
rNa$_v$1.6[7]	O88420[2]	agqCpeg(O)	qcpEgfq(O)	yieQrkt(N)	eqrKtir(N)	gidDmfn(O)	ldkEhpg(O)	dkeHpgs(N)	kehPgsg(O)
hNa$_v$1.7[5]	6J8G[1]	sgqCpeg(O)	qcpEgyt(O)	yieRkkt(N)	erkKtik(N)	ginDmfn(O)	pkkVhpg[NI]	kkvHpgs(N)	kvhPgss(O)
mNa$_v$1.7[7]	Q62205[2]	sgqCpeg(O)	qcpEgye(O)	yieKkkt(N)	ekkKtik(N)	ginDmfn(O)	pkkVhpg[NI]	kkvHpgs(N)	kvhPgss(O)
rNa$_v$1.7[7]	O08562[2]	sgqCpeg(O)	qcpEgyi(O)	yieKkkt(N)	ekkKtik(N)	ginDmfn(O)	pkkVhpg[NI]	kkvHpgs(N)	kvhPgss(O)
hNa$_v$1.8[7]	Q9Y5Y9[2]	sghCpdg(O)	hcpDgyi(O)	yldQkpt(N)	dqkPtvk[NI]	gidDmfn(O)	pnlPnsn[NI]	nlpNsng(O)	lpnSngt(O)
mNa$_v$1.8[7]	Q6QIY3[2]	aghCpnd(O)	hcpNdyv(O)	yleEkpr[NI]	eekPrvk[NI]	gidDmfn(O)	pnrPnsn[NI]	nrpNsng(O)	rpnSngs(O)
rNa$_v$1.8[7]	Q62968[2]	aghCpgg(O)	hcpGgyv[NI]	yleEkpr[NI]	eekPrvk[NI]	gidDmfn(O)	pnlPnsn[NI]	nlpNsng(O)	lpnSngs(O)
hNa$_v$1.9[7]	Q9UI33[2]	nsaCsiq(O)	acsIqye[NI]	hleNqpk(N)	enqPkiq[NI]	gidDifn(O)	rskEscn[NI]	skeScns[NI]	kesCnss[NI]
mNa$_v$1.9[7]	Q9R053[2]	rrsCpdg(O)	scpDgst(O)	nlpSrpq[NI]	psrPqve[NI]	gidDifn(O)	eskAscn[NI]	skaScns[NI]	kasCnss[NI]
rNa$_v$1.9[7]	O88457[2]	srpCpng(O)	pcpNgst(O)	nlpSrpq[NI]	psrPqve[NI]	gidDifn(O)	eakEhcn[NI]	akeHcns[NI]	kehCnss[NI]
PPI Id[4]		1b	2b	3b	4b	5b	6b	7b	8b
eeNa$_v$β1[5]	5XSY[1]	sckMrge[NI]	yfdRtlt(N)	acvEvds(O)	vdsDtea(O)	ckmRgev(N)	mgsKntf(N)	sngAcve(N)	gacVevd(N)
hNa$_v$β1[5]	6AGF[1]	sckRrse(N)	hvyRllf(N)	gcvEvds(O)	vdsEtea(O)	ckrRset(N)	ngsRgtk(N)	acgGcve(N)	ggcVevd(N)
mNa$_v$β1[7]	P97952[2]	sckRrse(N)	hvyRllf(N)	gcvEvds(O)	vdsDtea(O)	ckrRset(N)	ngsRgtk(N)	awgGcve(N)	ggcVevd(N)
rNa$_v$β1[7]	Q00954[2]	sckRrse(N)	hvyRllf(N)	gcvEvds(O)	vdsEtea(O)	ckrRset(N)	ngsRgtk(N)	awgGcve(N)	ggcVevd(N)
hNa$_v$β3[6]	4L1D[1]	scmKree(N)	nvsRefe(N)	vcvEvps(O)	vpsEtea(O)	cmkReev(N)	ngsKdlq(N)	cfpVcve(N)	pvcVevp(N)
mNa$_v$β3[7]	Q8BHK2	scmKree(N)	nvsRefe(N)	vcvEvps(O)	vpsEtea(O)	cmkReev(N)	ngsKdlq(N)	cfpVcve(N)	pvcVevp(N)
rNa$_v$β3[7]	Q9JK00[2]	scmKree(N)	nvsRefe(N)	vcvEvps(O)	vpsEtea(O)	cmkReev(N)	ngsKdlq(N)	cfpVcve(N)	pvcVevp(N)

[1]PDB code (http://www.rcsb.org/), [2]UniProt code (https://www.uniprot.org/), [3]PPI-Id for positions of computed polar interactions the residues in Na$_v$$\alpha$ (1 – 8), [4]Id for positions of computed polar interactions of the residues in Na$_v$β1 and Na$_v$β3 (1b – 8b) with respect to Na$_v$$\alpha$, templates ([5]Cryo-EM structures, [6]crystal structures), [7]models, [NI] no interaction; residues that form computed polar interactions (bold capital letters); amino acids adjacent in sequence to residues that form computed polar interactions (lowercase letters); positively and negatively charged residues (blue and red, respectively), polar residues (cyan), non-polar residues (orange).

TABLE S1 – alternative presentation. Symbols: YES means there is a contribution to the PPI. NO means there is none. See also, Table 1 in the manuscript, MS.

Isoform		Extracelular loop

	Codes of PDB or UniProt entries	S5 DI		S1-S2 DIII		S5 DIV	S6 DIV		
		Id of interactions							
		1	2	3	4	5	6	7	8
*eeNa$_v$1.4	5XSY[PDB]	NO	YES	YES	YES	YES	YES	YES	YES
hNa$_v$1.1	P35498A[UP]	YES	YES	YES	YES	YES	NO	YES	YES
mNa$_v$1.1	A2APX8	YES	YES	YES	YES	YES	NO	YES	YES
rNa$_v$1.1	P04774 [UP]	YES	YES	YES	YES	YES	NO	YES	YES
hNa$_v$1.2	6J8E[PDB]	YES	YES	YES	YES	YES	YES	YES	YES
mNa$_v$1.2	A0A0R5R P28 [UP]	YES	YES	YES	YES	YES	YES	YES	YES
rNa$_v$1.2	P04775 [UP]	YES	YES	YES	YES	YES	YES	YES	YES
hNa$_v$1.3	Q9NY46 [UP]	YES	YES	YES	YES	YES	NO	YES	YES
mNa$_v$1.3	A2AYES5 UP	YES	YES	YES	YES	YES	NO	YES	YES
rNa$_v$1.3	P08104 [UP]	YES	YES	YES	YES	YES	NO	YES	YES
hNa$_v$1.4	6AGF[PDB]	YES	YES	YES	YES	YES	YES	YES	YES
mNa$_v$1.4	Q9ER60 [UP]	YES	YES	YES	YES	YES	YES	YES	YES
rNa$_v$1.4	P15390 [UP]	YES	YES	YES	YES	YES	YES	YES	YES
hNa$_v$1.5	Q14524 [UP]	YES	YES	NO	YES	YES	NO	YES	YES
mNa$_v$1.5	Q9JJV9 [UP]	YES	YES	NO	YES	YES	NO	YES	YES
rNa$_v$1.5	6U70 [PDB]	YES	YES	NO	YES	YES	NO	YES	YES
hNa$_v$1.6	Q9UQD0 [UP]	YES	YES	YES	YES	YES	YES	YES	YES
mNa$_v$1.6	Q9WTU3 [UP]	YES	YES	YES	YES	YES	YES	YES	YES
rNa$_v$1.6	O88420 [UP]	YES	YES	YES	YES	YES	YES	YES	YES
hNa$_v$1.7	6J8G[PDB]	YES	YES	YES	YES	YES	NO	YES	YES
mNa$_v$1.7	Q62205 [UP]	YES	YES	YES	YES	YES	NO	YES	YES
rNa$_v$1.7	O08562 [UP]	YES	YES	YES	YES	YES	NO	YES	YES
hNa$_v$1.8	Q9Y5Y9 [UP]	YES	YES	YES	NO	YES	NO	YES	YES
mNa$_v$1.8	Q6QIY3 [UP]	YES	YES	NO	NO	YES	NO	YES	YES
rNa$_v$1.8	Q62968 [UP]	YES	NO	NO	NO	YES	NO	YES	YES
hNa$_v$1.9	Q9UI33 [UP]	YES	NO	YES	NO	YES	NO	NO	NO
mNa$_v$1.9	Q9R053 [UP]	YES	YES	NO	NO	YES	NO	NO	NO
rNa$_v$1.9	O88457 [UP]	YES	YES	NO	NO	YES	NO	NO	NO

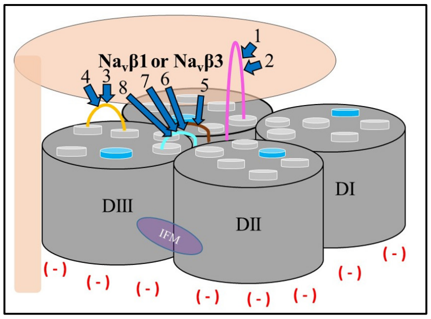

Blue = H-Bond

Yellow = Salt Bridge

Red = No interaction

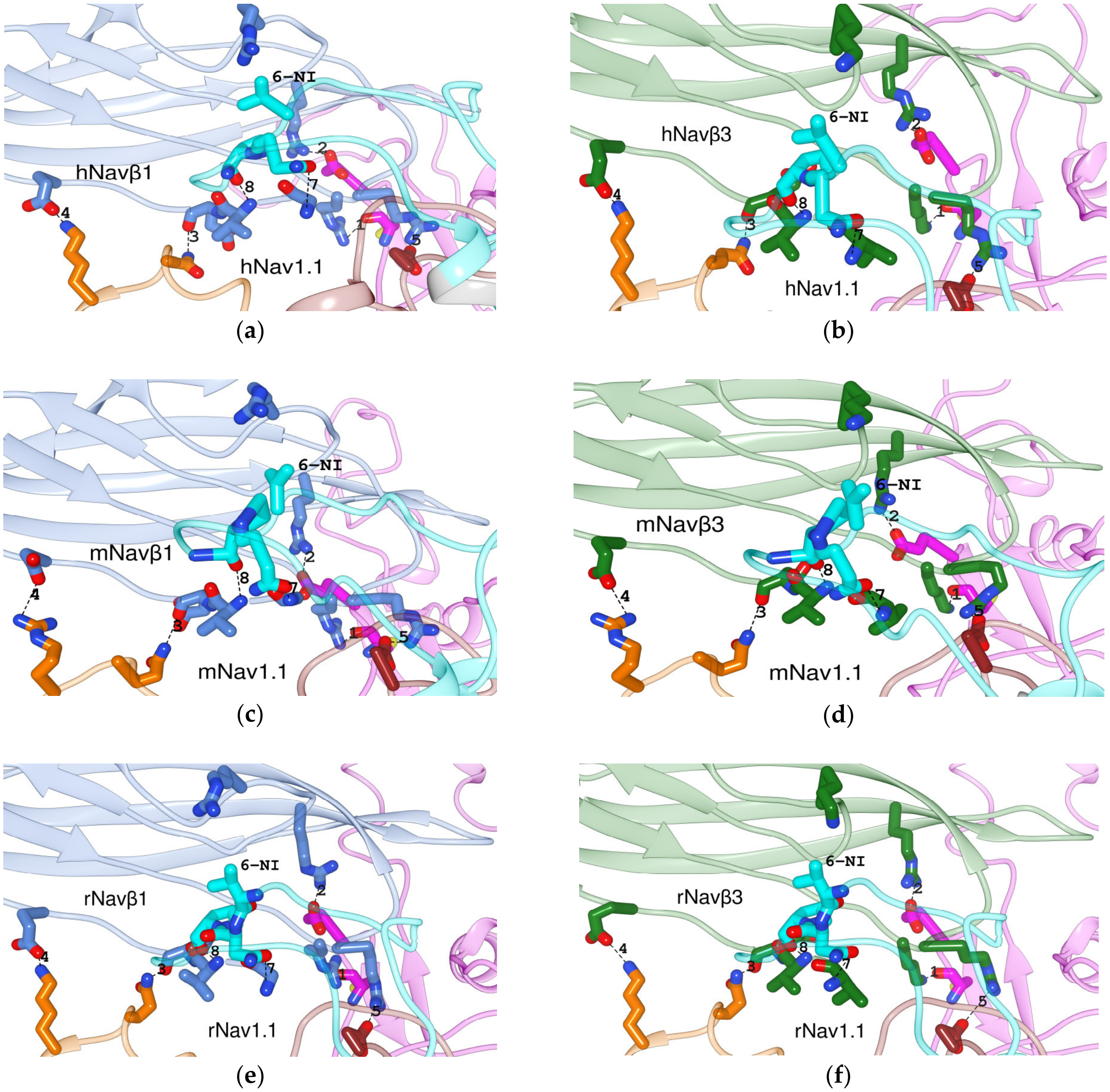

Figure S1. PPIs of the three h,m,rNa$_v$1.1 isoforms in complex with Na$_v$β1 and Na$_v$β3. (**a**, **b**) complexes of human; (**c**, **d**) mouse; (**e**, **f**) rat; residues that do not participate in the interface: gray; S5 DI: magenta; S1-S2 DIII: orange; S5 DIV: brown; S6 DIV: cyan; Na$_v$β1: cornflower blue; Na$_v$β3: forest green; numbers: PPI Id according to the Table S1; NI: no interaction.

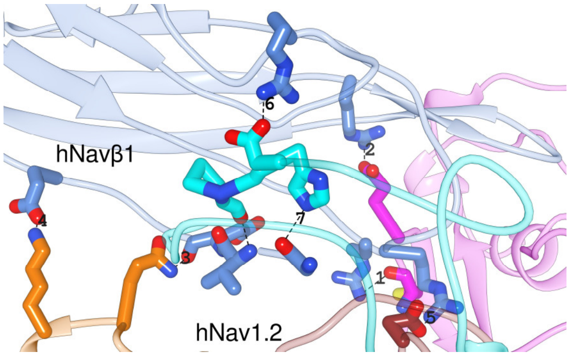

(**a**)

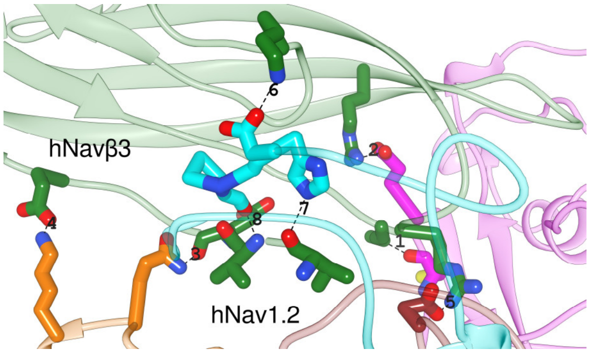

(**b**)

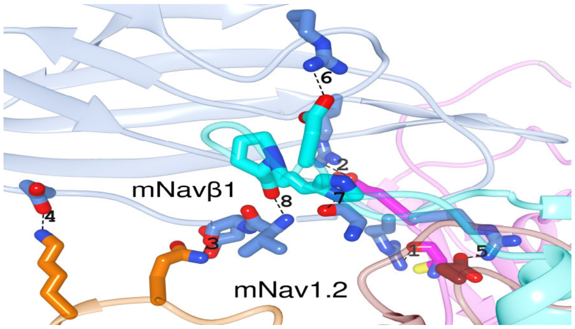

(**c**)

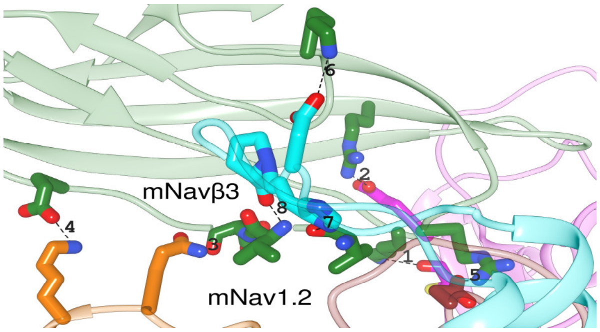

(**d**)

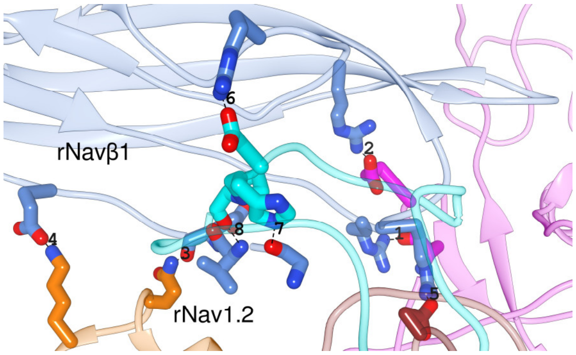

(**e**)

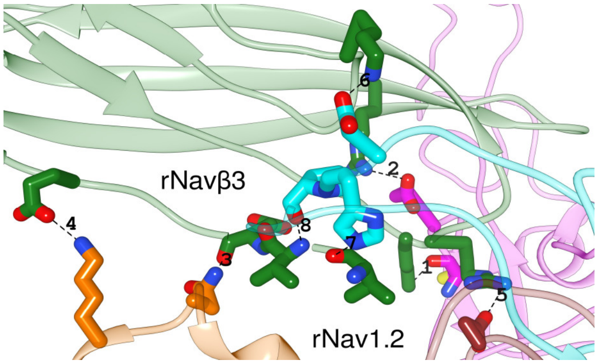

(**f**)

Figure S2. PPIs of the three h,m,rNa$_v$1.2 isoforms in complex with Na$_v$β1 and Na$_v$β3. (**a**, **b**) complexes of human; (**c**, **d**) mouse; (**e**, **f**) rat; residues that do not participate in the interface: gray; S5 DI: magenta; S1-S2 DIII: orange; S5 DIV: brown; S6 DIV: cyan; Na$_v$β1: cornflower blue; Na$_v$β3: forest green; numbers: PPI Id according to the Table S1; NI: no interaction.

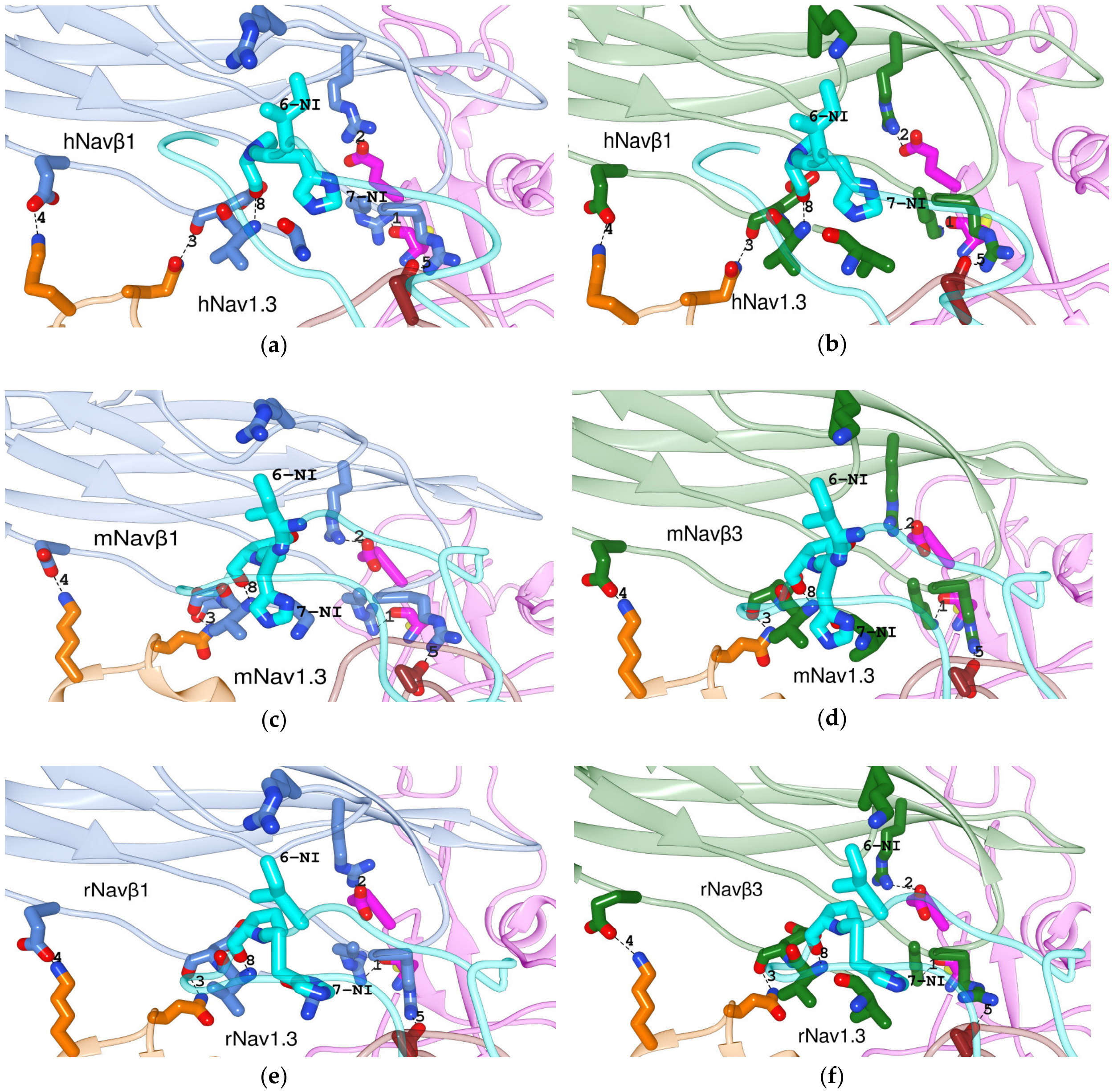

Figure S3. PPIs of the three $Na_v1.3$ isoforms in complex with $Na_v\beta1$ and $Na_v\beta3$. (**a**, **b**) complexes of human; (**c**, **d**) mouse; (**e**, **f**) rat; residues that do not participate in the interface: gray; S5 DI: magenta; S1-S2 DIII: orange; S5 DIV: brown; S6 DIV: cyan; $Na_v\beta1$: cornflower blue; $Na_v\beta3$: forest green; numbers: PPI Id according to the Table S1; NI: no interaction.

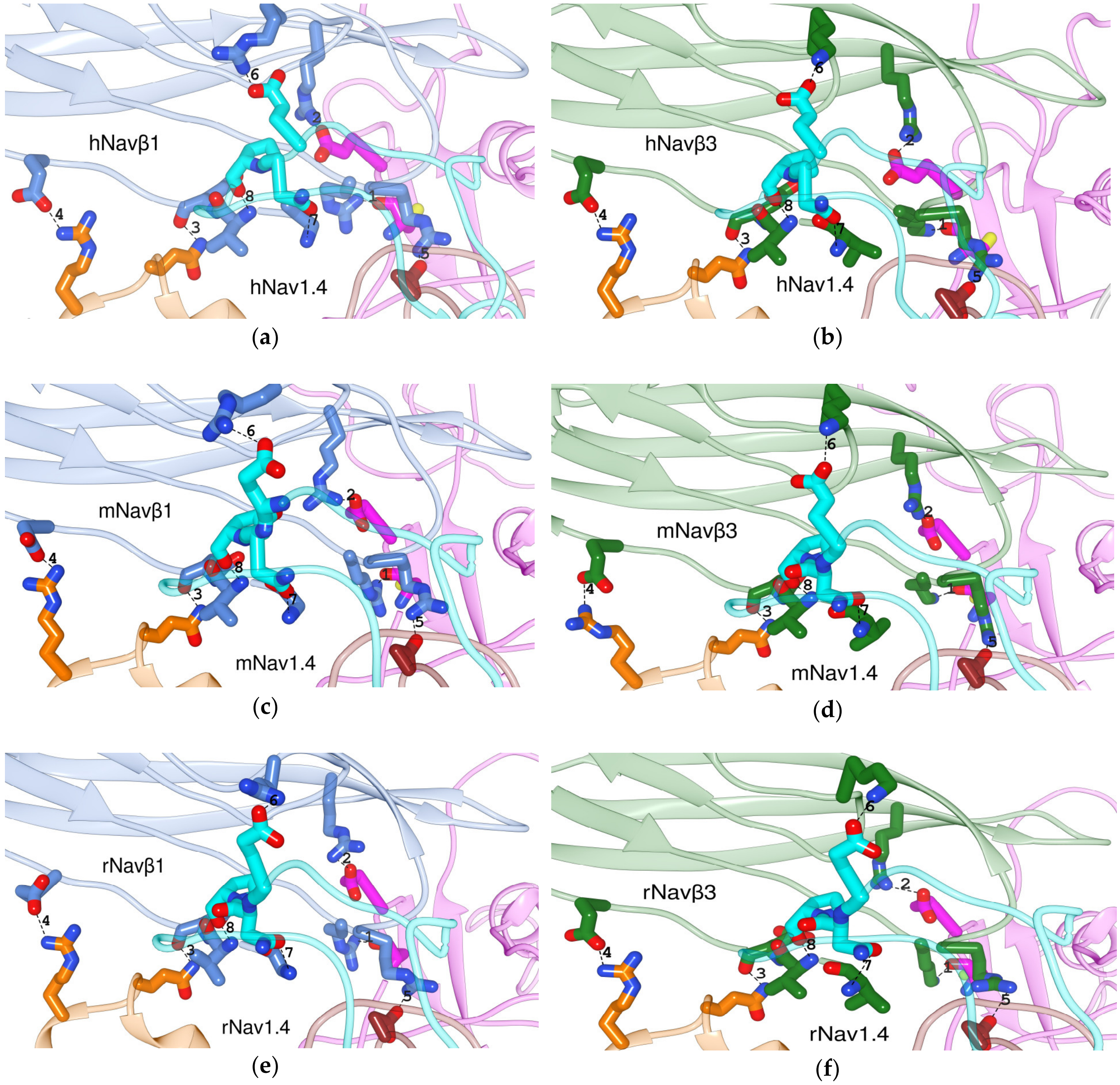

Figure S4. PPIs of the three h,m,rNa$_v$1.4 isoforms in complex with Na$_v$β1 and Na$_v$β3. (**a**, **b**) complexes of human; (**c**, **d**) mouse; (**e**, **f**) rat; residues that do not participate in the interface: gray; S5 DI: magenta; S1-S2 DIII: orange; S5 DIV: brown; S6 DIV: cyan; Na$_v$β1: cornflower blue; Na$_v$β3: forest green; numbers: PPI Id according to the Table S1; NI: no interaction.

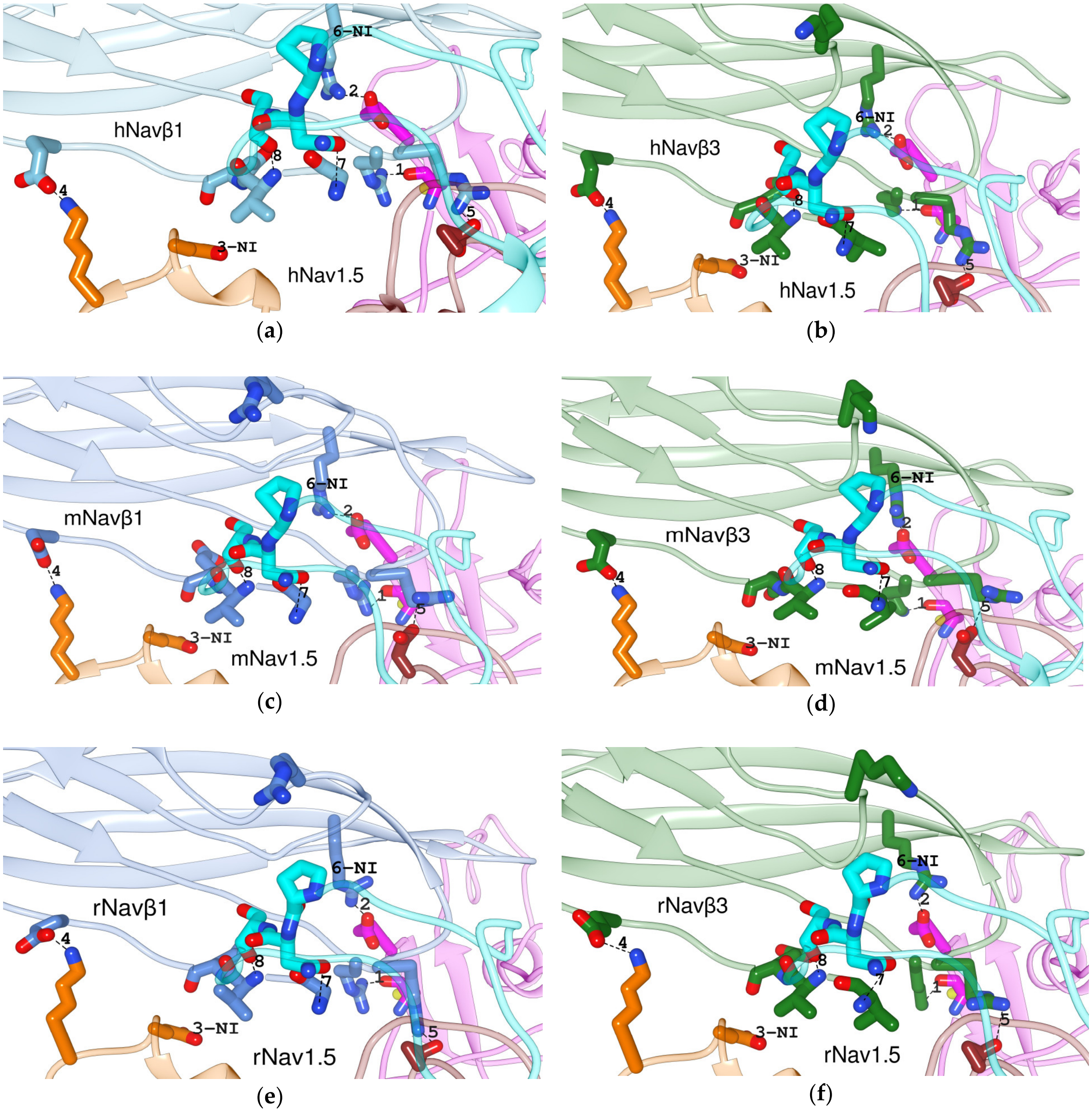

Figure S5. PPIs of the three h,m,rNa$_v$1.5 isoforms in complex with Na$_v$β1 and Na$_v$β3. (**a**, **b**) complexes of human; (**c**, **d**) mouse; (**e**, **f**) rat; residues that do not participate in the interface: gray; S5 DI: magenta; S1-S2 DIII: orange; S5 DIV: brown; S6 DIV: cyan; Na$_v$β1: cornflower blue; Na$_v$β3: forest green; numbers: PPI Id according to the Table S1; NI: no interaction.

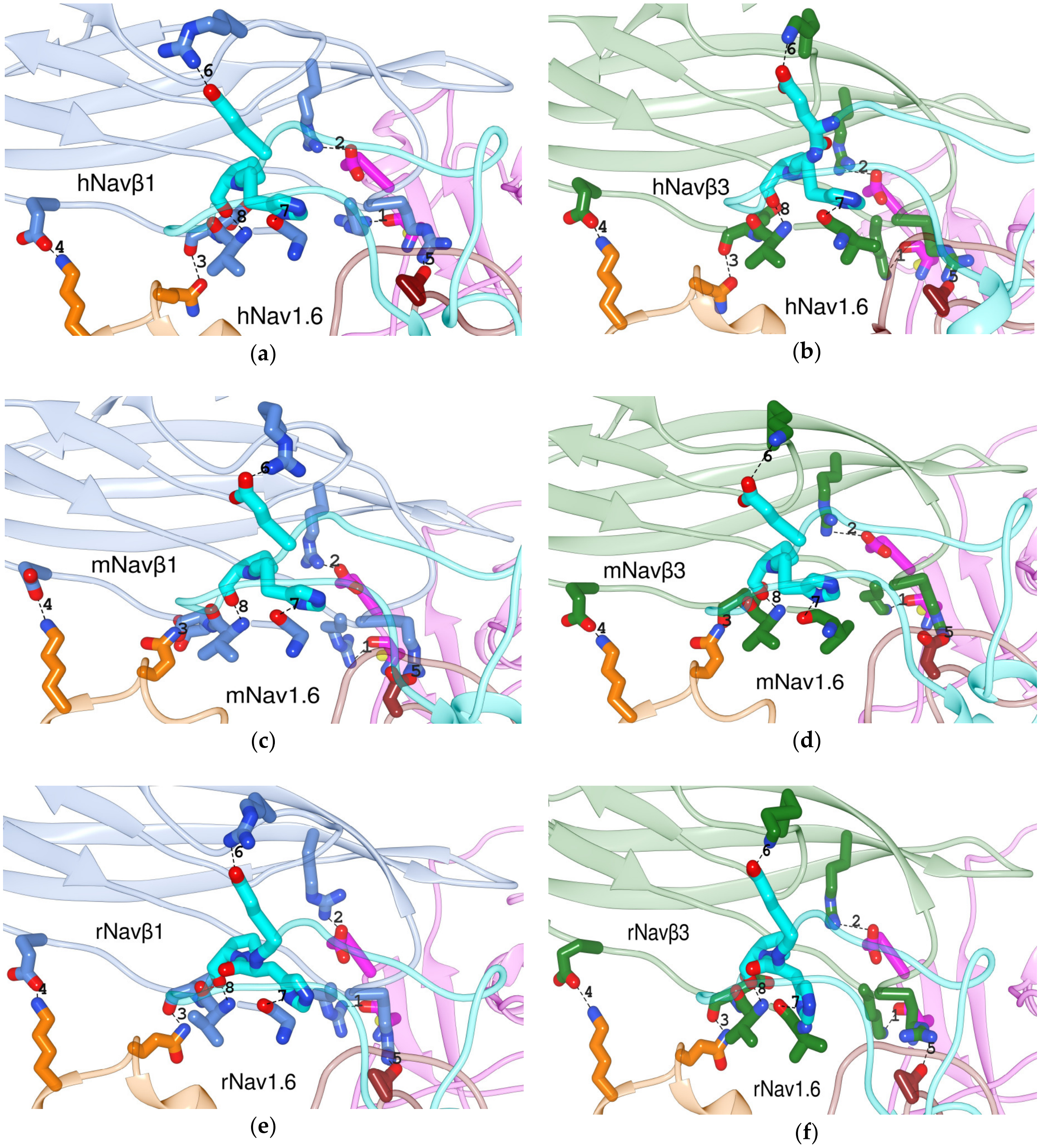

Figure S6. PPIs of the three h,m,rNa$_v$1.6 isoforms in complex with Na$_v$β1 and Na$_v$β3. (**a**, **b**) complexes of human; (**c**, **d**) mouse; (**e**, **f**) rat; residues that do not participate in the interface: gray; S5 DI: magenta; S1-S2 DIII: orange; S5 DIV: brown; S6 DIV: cyan; Na$_v$β1: cornflower blue; Na$_v$β3: forest green; numbers: PPI Id according to the Table S1; NI: no interaction.

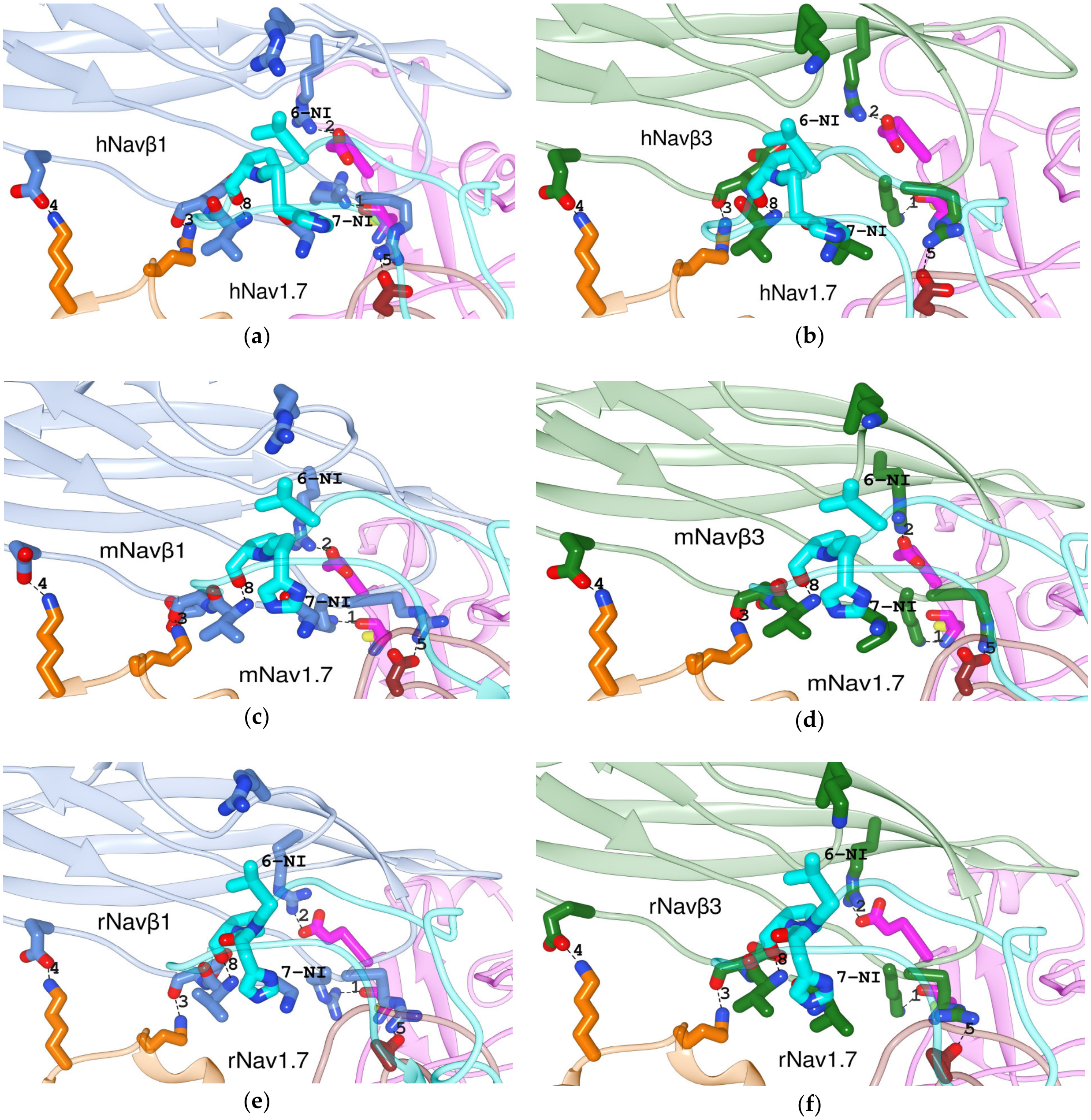

Figure S7. PPIs of the three h,m,rNa_v1.7 isoforms in complex with Na_vβ1 and Na_vβ3. (**a**, **b**) complexes of human; (**c**, **d**) mouse; (**e**, **f**) rat; residues that do not participate in the interface: gray; S5 DI: magenta; S1-S2 DIII: orange; S5 DIV: brown; S6 DIV: cyan; Na_vβ1: cornflower blue; Na_vβ3: forest green; numbers: PPI Id according to the Table S1; NI: no interaction.

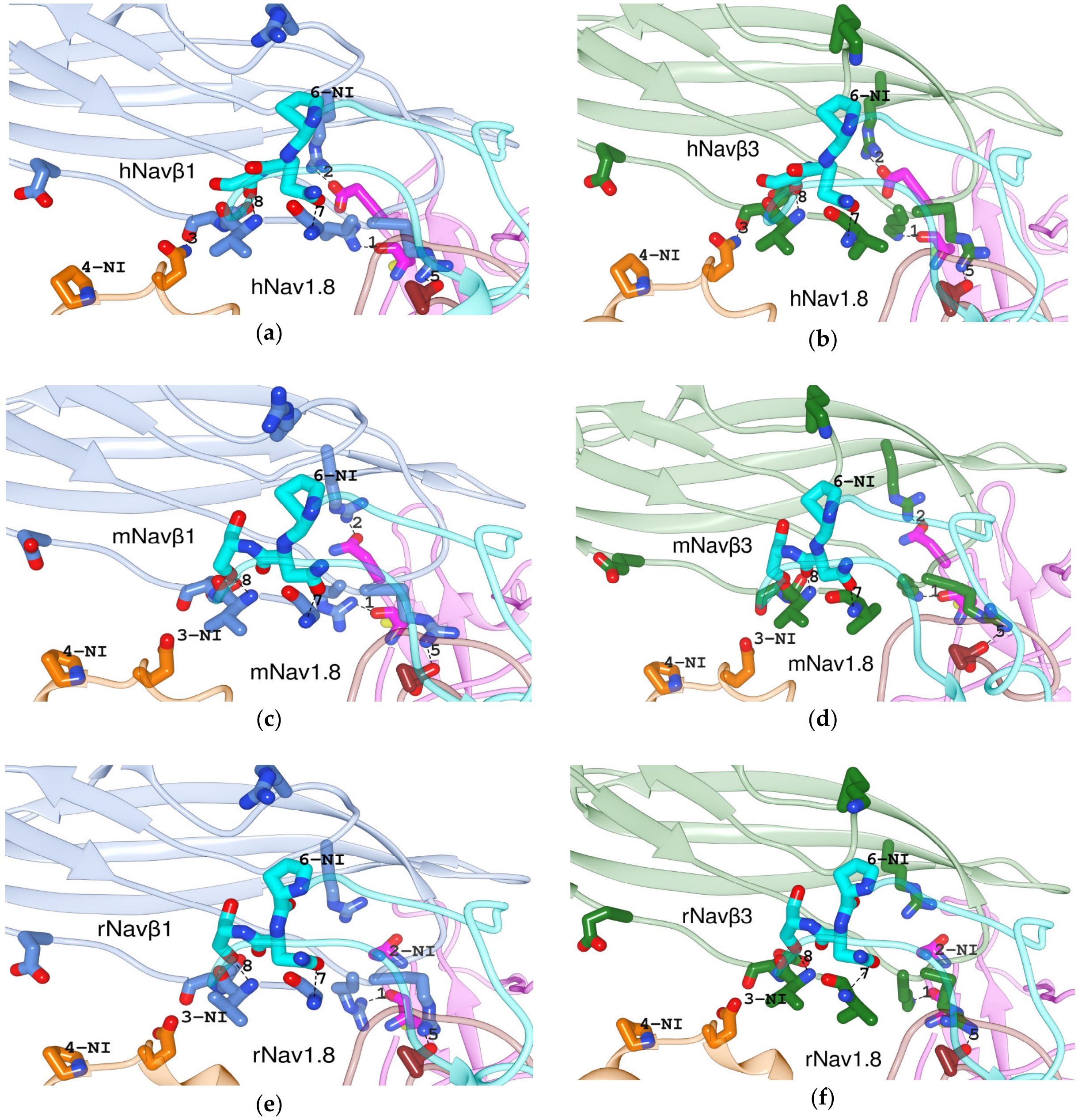

Figure S8. PPIs of the three h,m,rNa$_v$1.8 isoforms in complex with Na$_v$β1 and Na$_v$β3. (**a**, **b**) complexes of human; (**c**, **d**) mouse; (**e**, **f**) rat; residues that do not participate in the interface: gray; S5 DI: magenta; S1-S2 DIII: orange; S5 DIV: brown; S6 DIV: cyan; Na$_v$β1: cornflower blue; Na$_v$β3: forest green; numbers: PPI Id according to the Table S1; NI: no interaction.

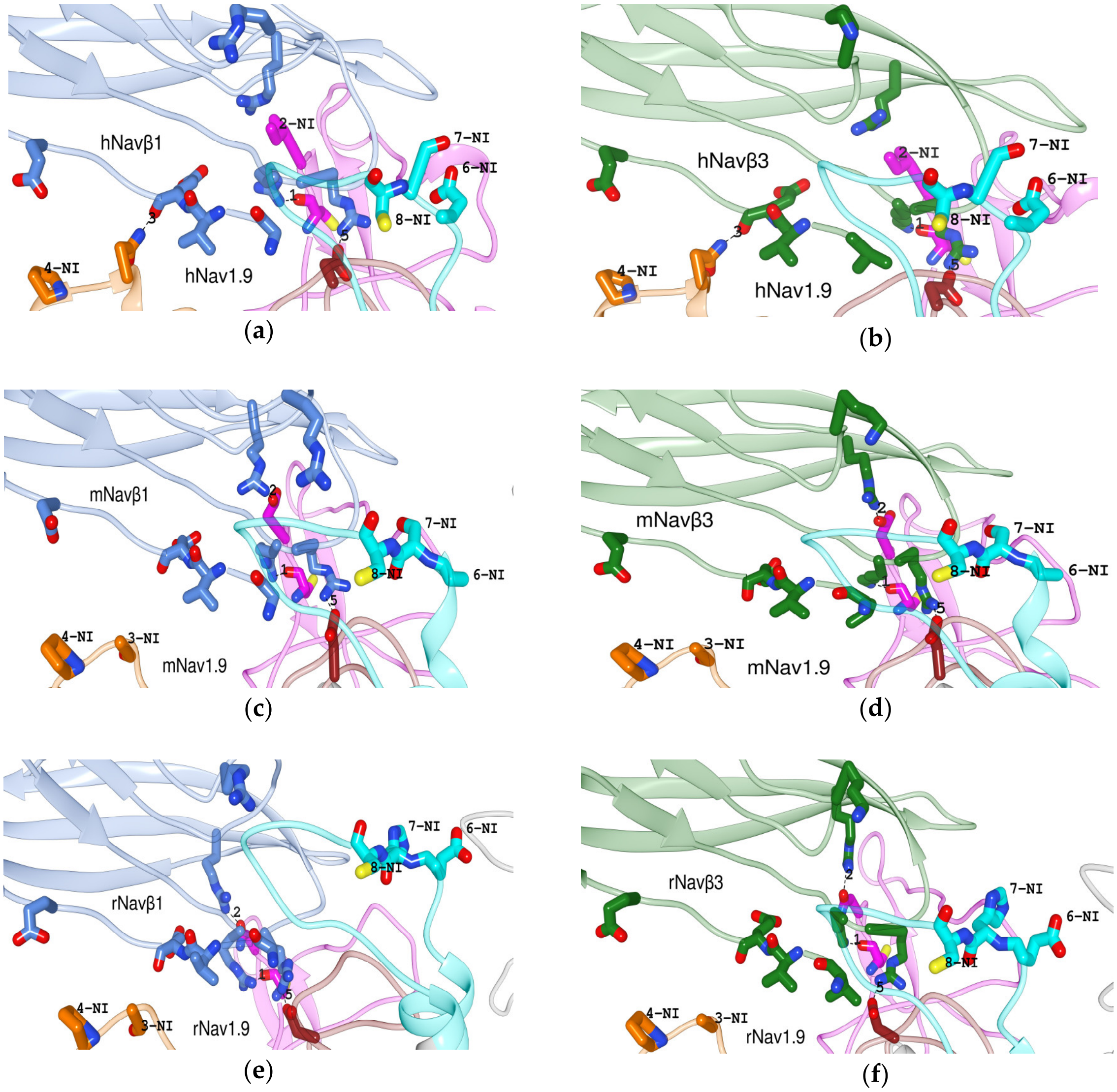

Figure S9. PPIs of the three h,m,rNa_v1.9 isoforms in complex with Na_vβ1 and Na_vβ3. (**a**, **b**) complexes of human; (**c**, **d**) mouse; (**e**, **f**) rat; residues that do not participate in the interface: gray; S5 DI: magenta; S1-S2 DIII: orange; S5 DIV: brown; S6 DIV: cyan; Na_vβ1: cornflower blue; Na_vβ3: forest green; numbers: PPI Id according to the Table S1; NI: no interaction.

Table S2. Residues of contact of Na_vs S4 DIII with the Na_vβ1 and Na_vβ3 subunits

**Na_vα**			**Na_vβ**					
**Isoform**	**Code**	**[1]Residues S4 DIII**	**Subunit**	**Code**	**[2]Residues**	**Subunit**	**Code**	**[2]Residues**
hNa_v1.1	[4]P35498	gai**K**sl**R**tlr						
hNa_v1.2	[3]6J8E	gai**K**sl**R**tlr	hNa_vβ1	[3]6AGF	mtf**K**ilc	hNa_vβ3	[3]4L1D	npm**K**lrc
hNa_v1.3	[4]Q9NY46	gai**K**sl**R**tlr						

hNa$_v$1.4	[3]6AGF	gpi**K**sl**R**tlr						
hNa$_v$1.5	[4]Q14524	gpi**K**sl**R**tlr						
hNa$_v$1.6	[4]Q9UQD0	gai**K**sl**R**tlr						
hNa$_v$1.7	[3]6J8G	gpi**K**sl**R**tlr						
hNa$_v$1.8	[4]Q9Y5Y9	api**K**al**R**tlr						
hNa$_v$1.9	[4]Q9UI33	mel**K**sf**R**tlr						
mNa$_v$1.1	[4]A2APX8	gai**K**sl**R**tlr						
mNa$_v$1.2	[4]B1AWN6	gai**K**sl**R**tlr						
mNa$_v$1.3	[4]A2ASI5	gai**K**sl**R**tlr						
mNa$_v$1.4	[4]Q9ER60	gpi**K**sl**R**tlr						
mNa$_v$1.5	[4]Q9JJV9	gpi**K**sl**R**tlr	mNa$_v$β1	[4]P97952	mtf**K**ilc	mNa$_v$β3	[4]Q8BHK2	nsm**K**lrc
mNa$_v$1.6	[4]Q9WTU3	gai**K**sl**R**tlr						
mNa$_v$1.7	[4]Q62205	gpi**K**sl**R**tlr						
mNa$_v$1.8	[4]Q6QIY3	asi**K**al**R**tlr						
mNa$_v$1.9	[4]Q9R053	pnl**K**sf**R**nlr						
rNa$_v$1.1	[4]P04774	gai**K**sl**R**tlr						
rNa$_v$1.2	[4]P04775	gai**K**sl**R**tlr						
rNa$_v$1.3	[4]P08104	gai**K**sl**R**tlr						
rNa$_v$1.4	[4]P15390	gpi**K**sl**R**tlr						
rNa$_v$1.5	[4]P15389	gpi**K**sl**R**tlr	rNa$_v$β1	[4]Q00954	mtf**K**ilc	rNa$_v$β3	[4]Q9JK00	npm**K**lrc
rNa$_v$1.6	[4]O88420	gai**K**sl**R**tlr						
rNa$_v$1.7	[4]O08562	gpi**K**sl**R**tlr						
rNa$_v$1.8	[4]Q62968	asi**K**al**R**tlr						
rNa$_v$1.9	[4]O88457	psl**K**sf**R**tlr						

[1]S4 DIII residues that interact with Na$_v$β1 and Na$_v$β3 subunits; [2]Residues of Na$_v$β1 and Na$_v$β3 subunits that interact with S4 DIII; [3]PDB code: http://www.rcsb.org/; [4]UniProt code: https://www.uniprot.org/. Of note, table is in black&white. The interacting amino acids are in bold face and capital letters, e.g. K and R.

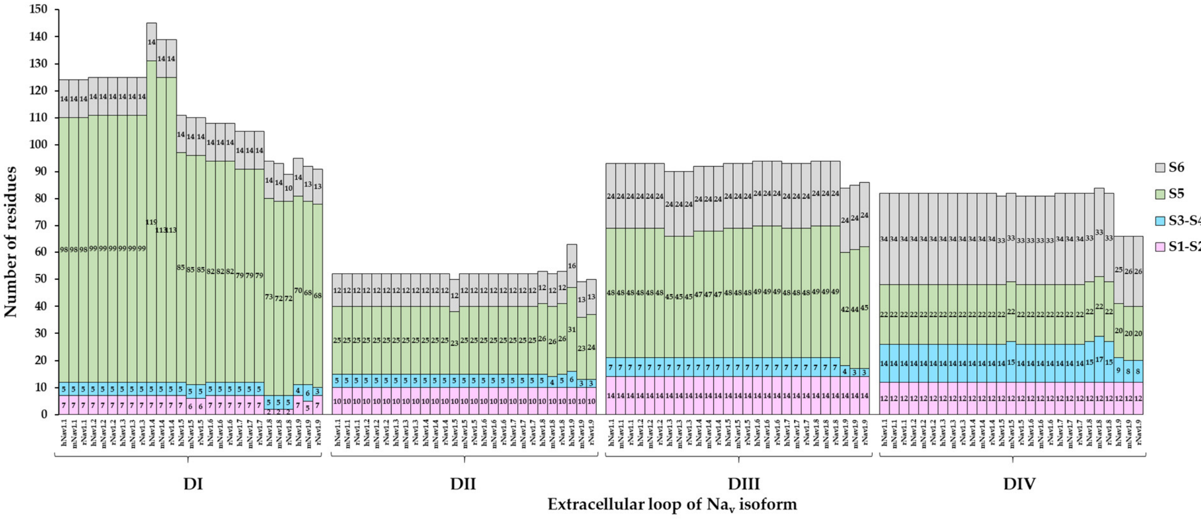

Figure S10. Length of extracellular loops of the Na_vs; extracellular loops: S1-S2, S3-S4, S5 and S6.

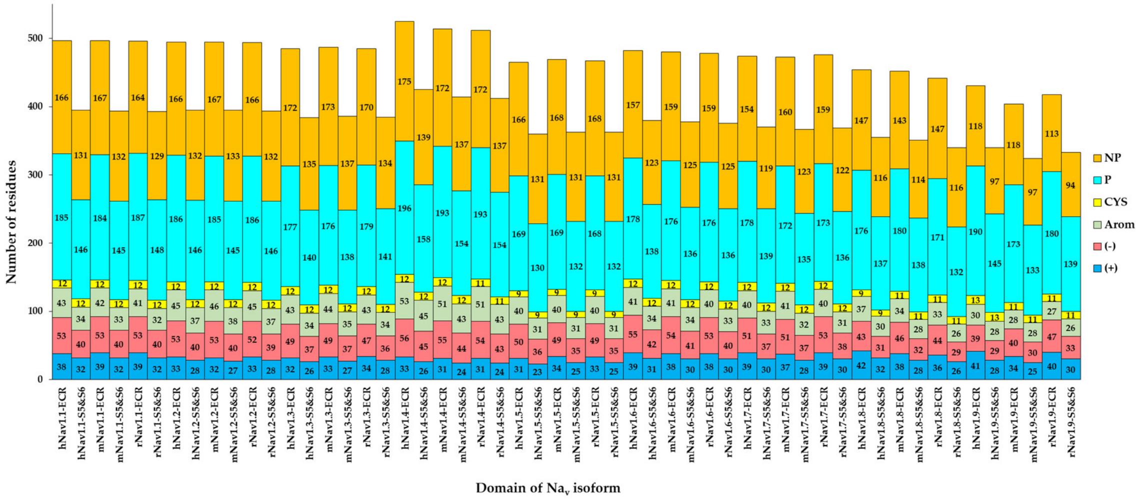

(**a**)

Figure S11. Properties of the residues of the extracellular region of the Na_v; (**a**) number of residues; ECR: extracellular region; S5 & S6: S5 and S6 extracellular loops; NP: nonpolar, P: polar; CYS: cysteines; Arom: aromatic; (-): negatively charged; (+): positively charged. The polar and non-polar residues represent the total residues of the analyzed region.

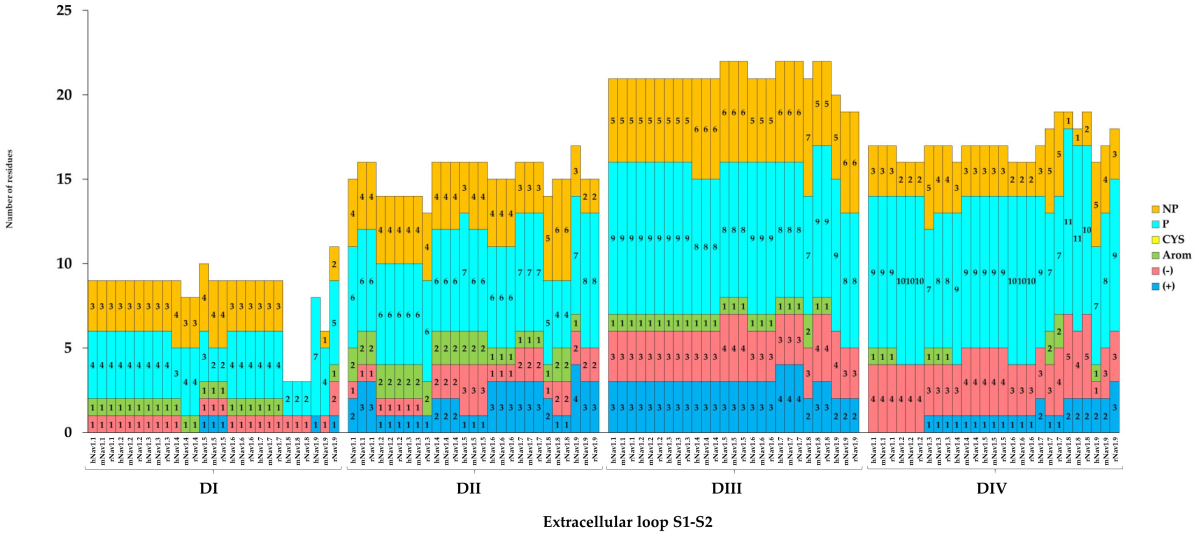
25
20
15
10
5
0
Number of residues
NP
P
CYS
Arom
(-)
(+)
DI
DII
DIII
DIV
Extracellular loop S1-S2

(**a**)

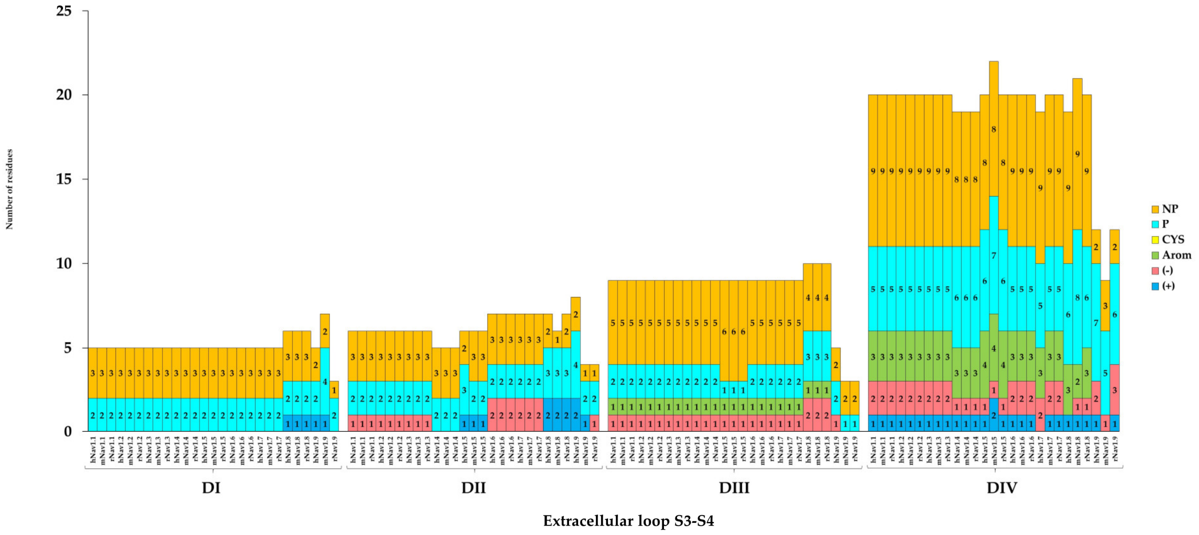
25
20
15
10
5
0
Number of residues
NP
P
CYS
Arom
(-)
(+)
DI
DII
DIII
DIV
Extracellular loop S3-S4

(**b**)

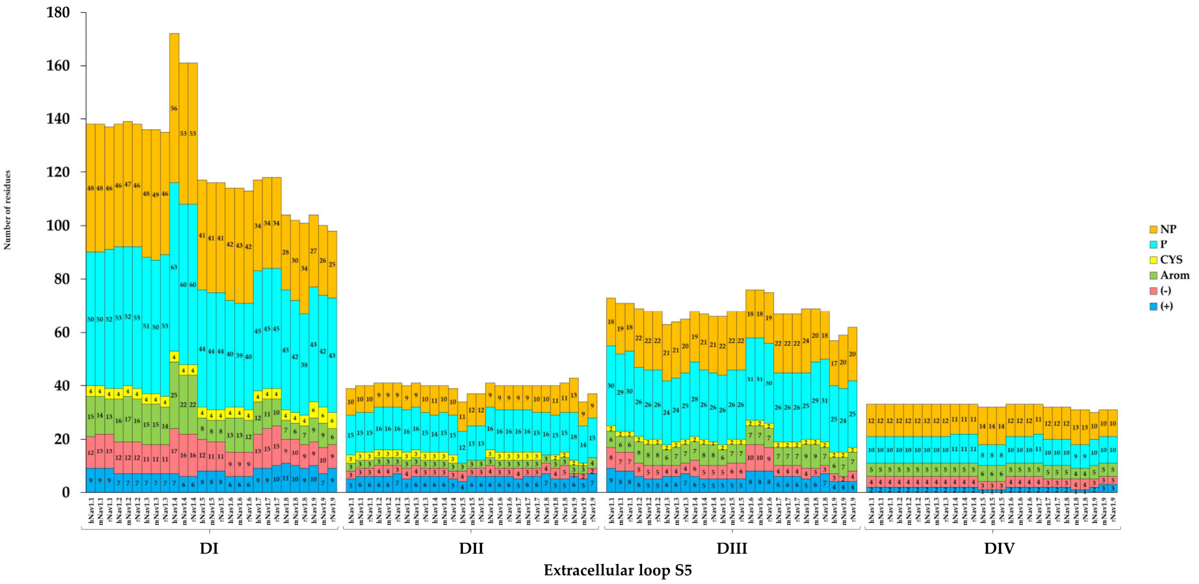

(**c**)

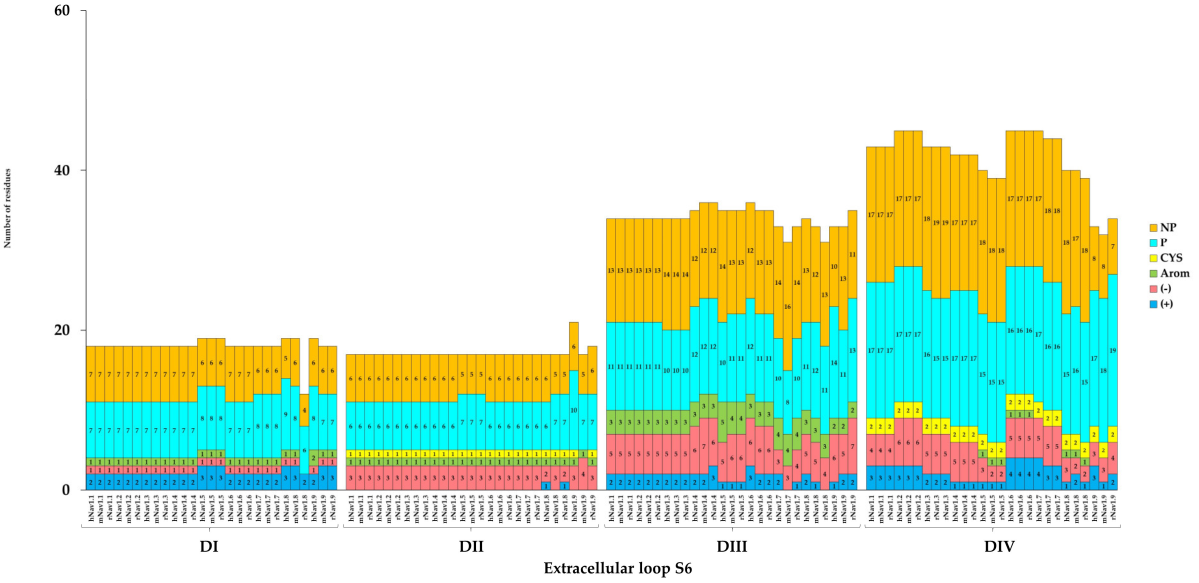

(**d**)

Figure S12. Properties of residues of Naᵥ extracellular loops; (**a**) S1-S2 extracellular loop; (**b**) S3-S4 extracellular loop; (**c**) S5 extracellular loop; (**d**) S6 extracellular loop; NP: nonpolar, P: polar, CYS: cysteines; Arom: aromatic; (-): negatively charged; (+): positively charged. The polar and non-polar residues represent the total residues of the analyzed region.

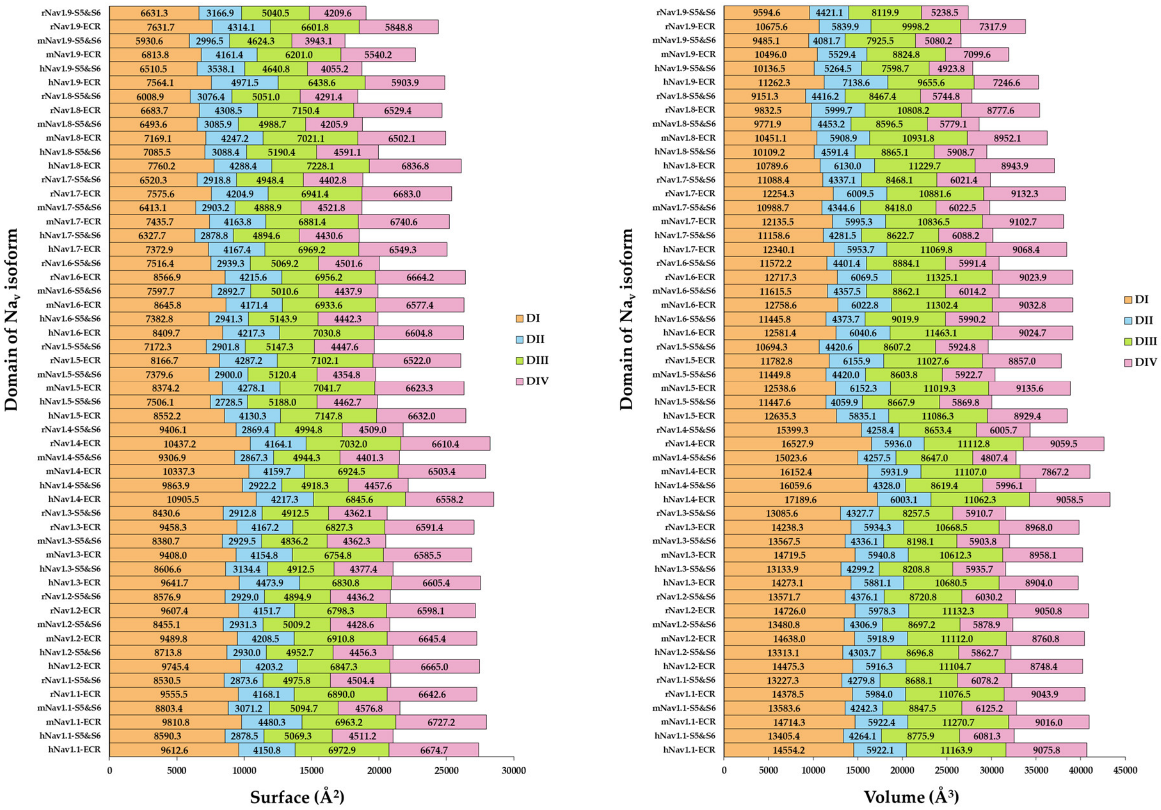

(**a**) (**b**)

Figure S13. Surface and volume properties of ECR and S5 and S6 extracellular loops (ECLs) of the Na$_v$s (**a**) SAA and (**b**) molecular volume; S5 & S6: S5 and S6 extracellular loops; DI – DIV: domains I – IV, respectively.

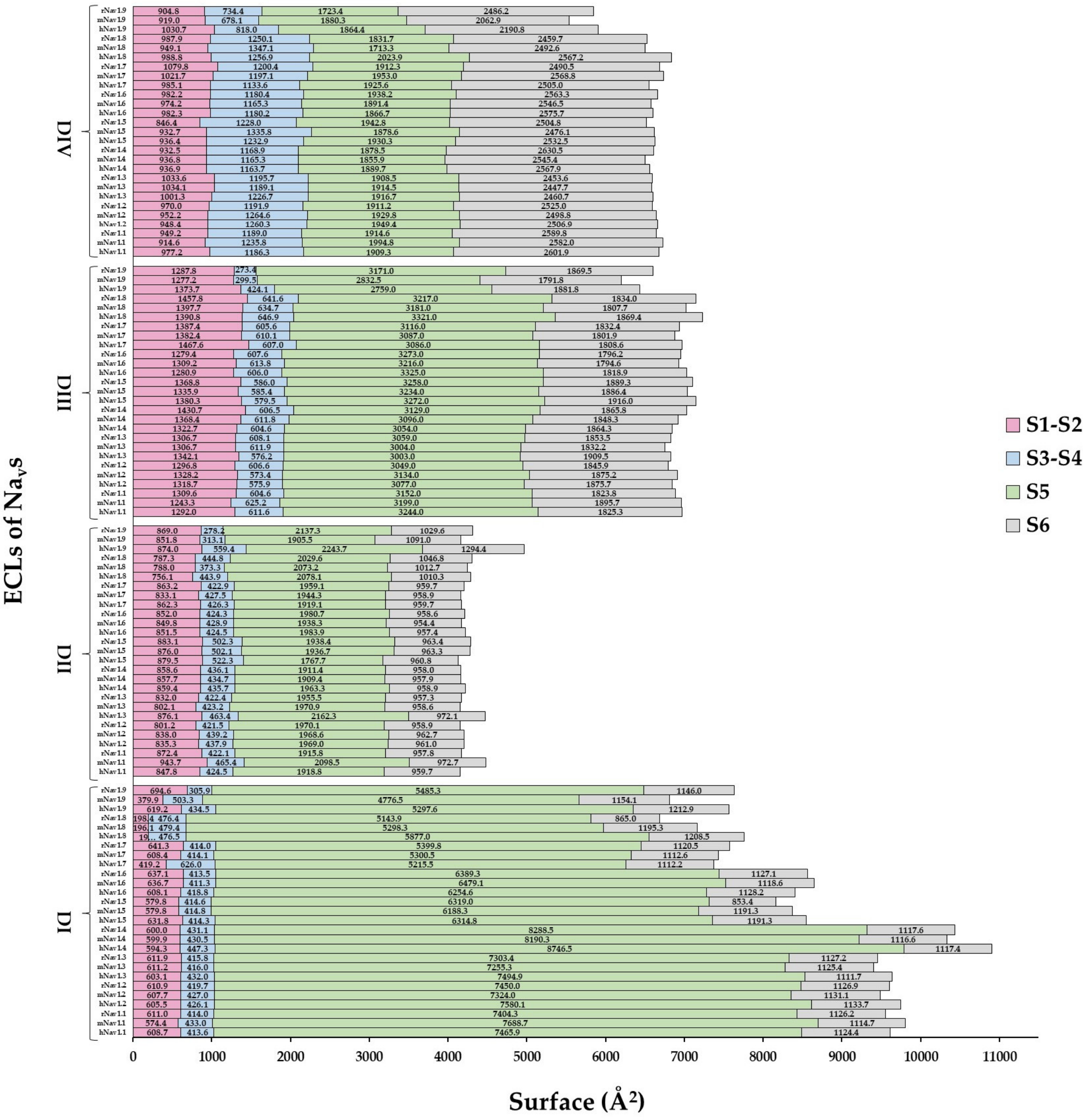

Figure S14. SAA of ECLs of the Navs; DI – DIV: domains I – IV, respectively.

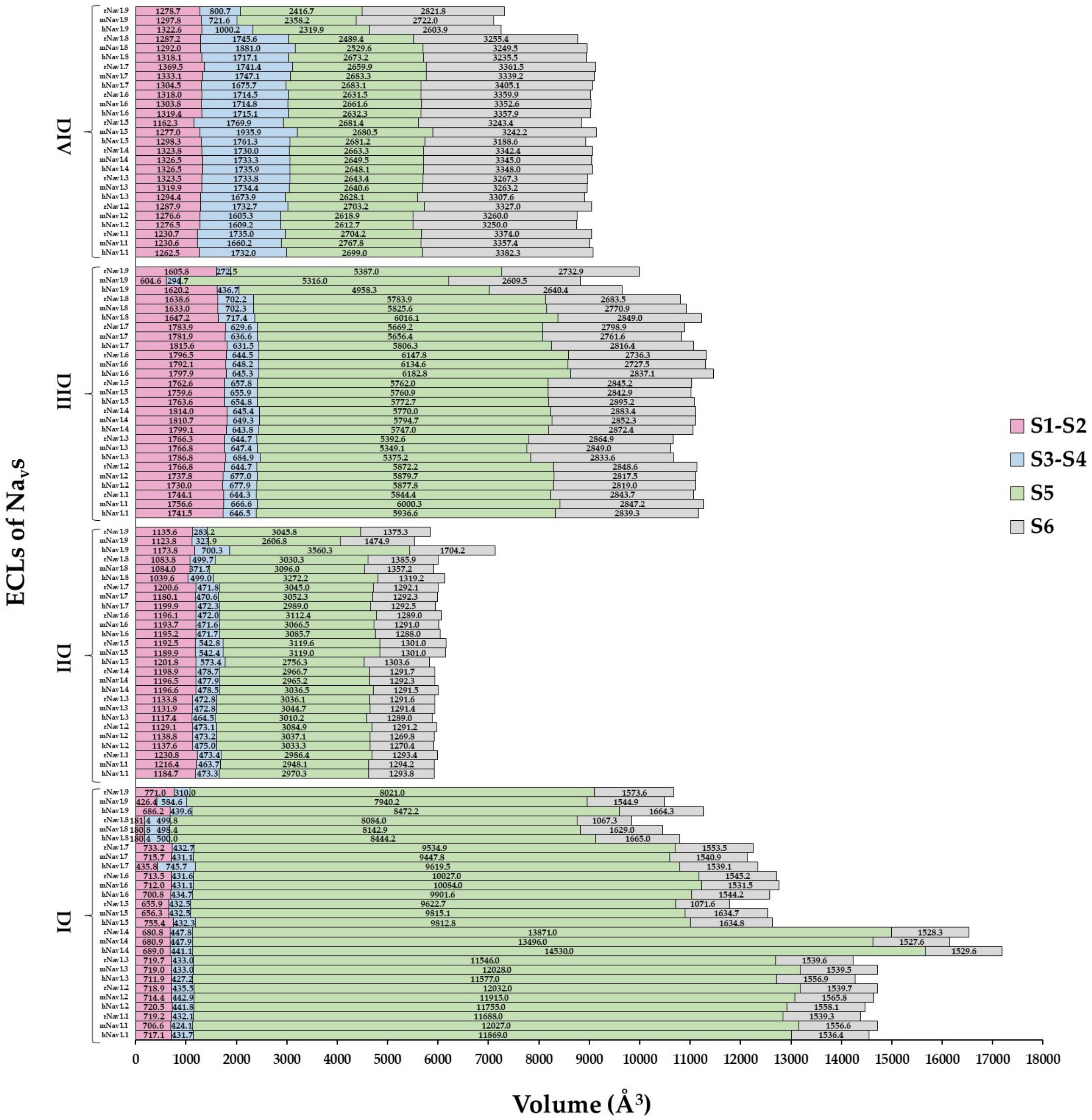

Figure S15. Molecular volume of ECLs of the Na$_V$s; DI – DIV: domains I – IV, respectively.

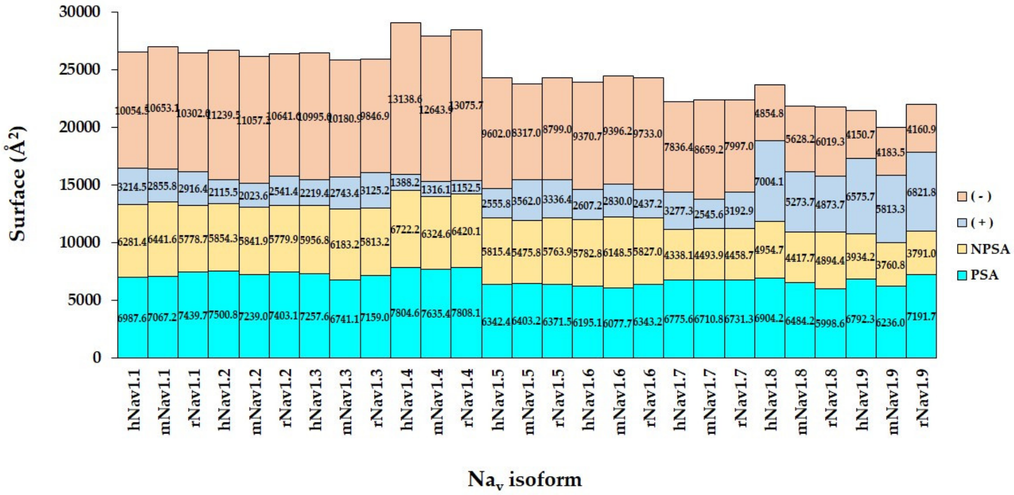

Figure S16. Properties of total SAA of IF-ECLs of Navs; PSA: Polar surface area; NPSA: non-polar surface area; (+): P-MEPS; (-): N-MEPS. The PSA and NPSA represents the total area of the analyzed region.

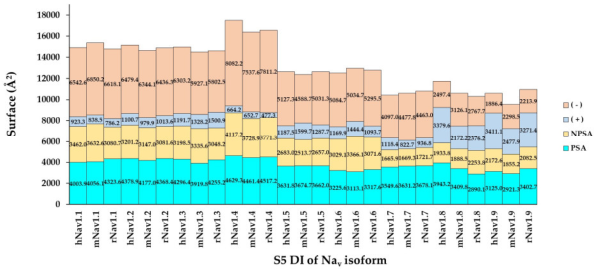

(**a**)

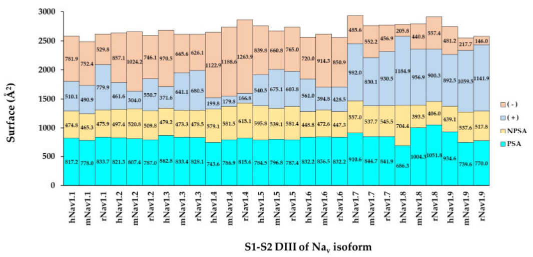

(**b**)

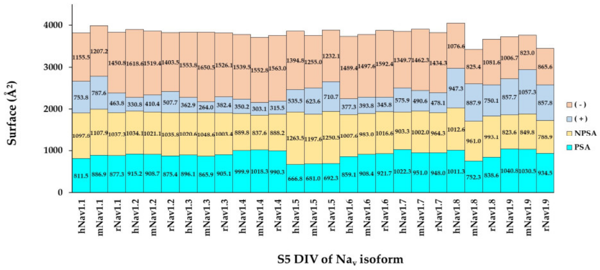

(**c**)

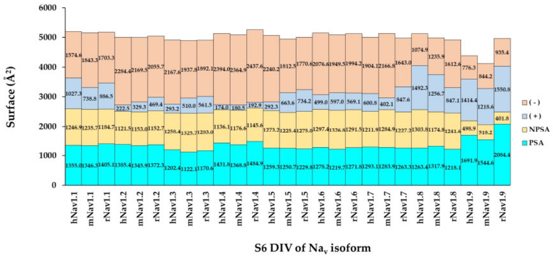

(**d**)

Figure S17. Properties of SAA of IF-ECLs of Nav; (**a**) S5 DI; (**b**) S1-S2 DIII (**c**) S5 DIV; (**d**) S6 DIV; PSA: Polar surface area; NPSA non-polar surface area; (+): P-MEPS; (-): N-MEPS. The PSA and NPSA represents the total area of the analyzed region.

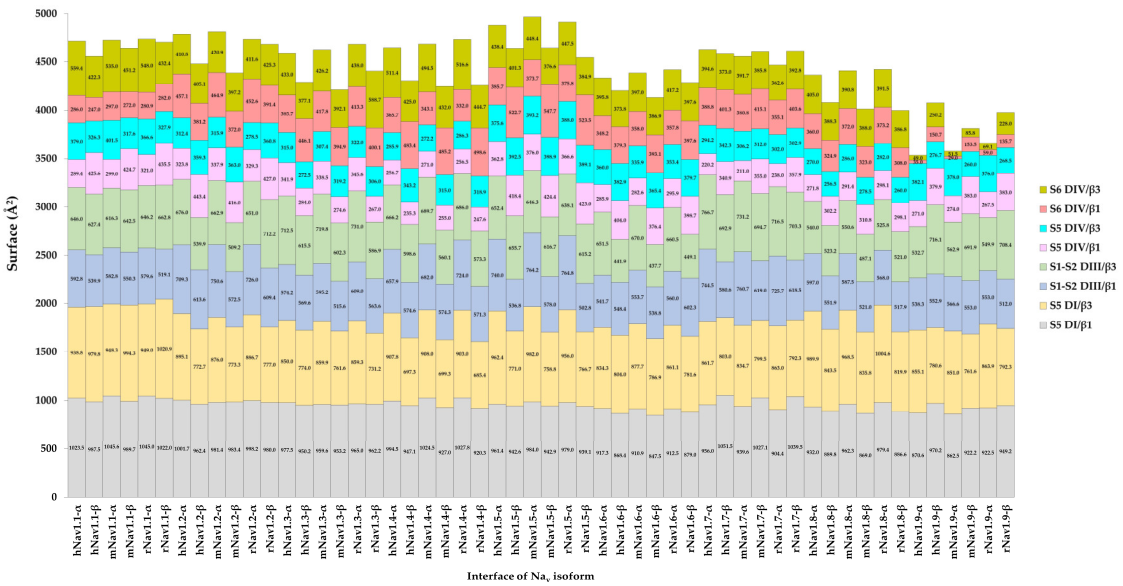

Figure S18. Total area of the atoms that form at the Naᵥα/Naᵥβ interface; α: area of the atoms in the α subunit that participate in the interface; β: area of the atoms in the β subunit that participate in the interface

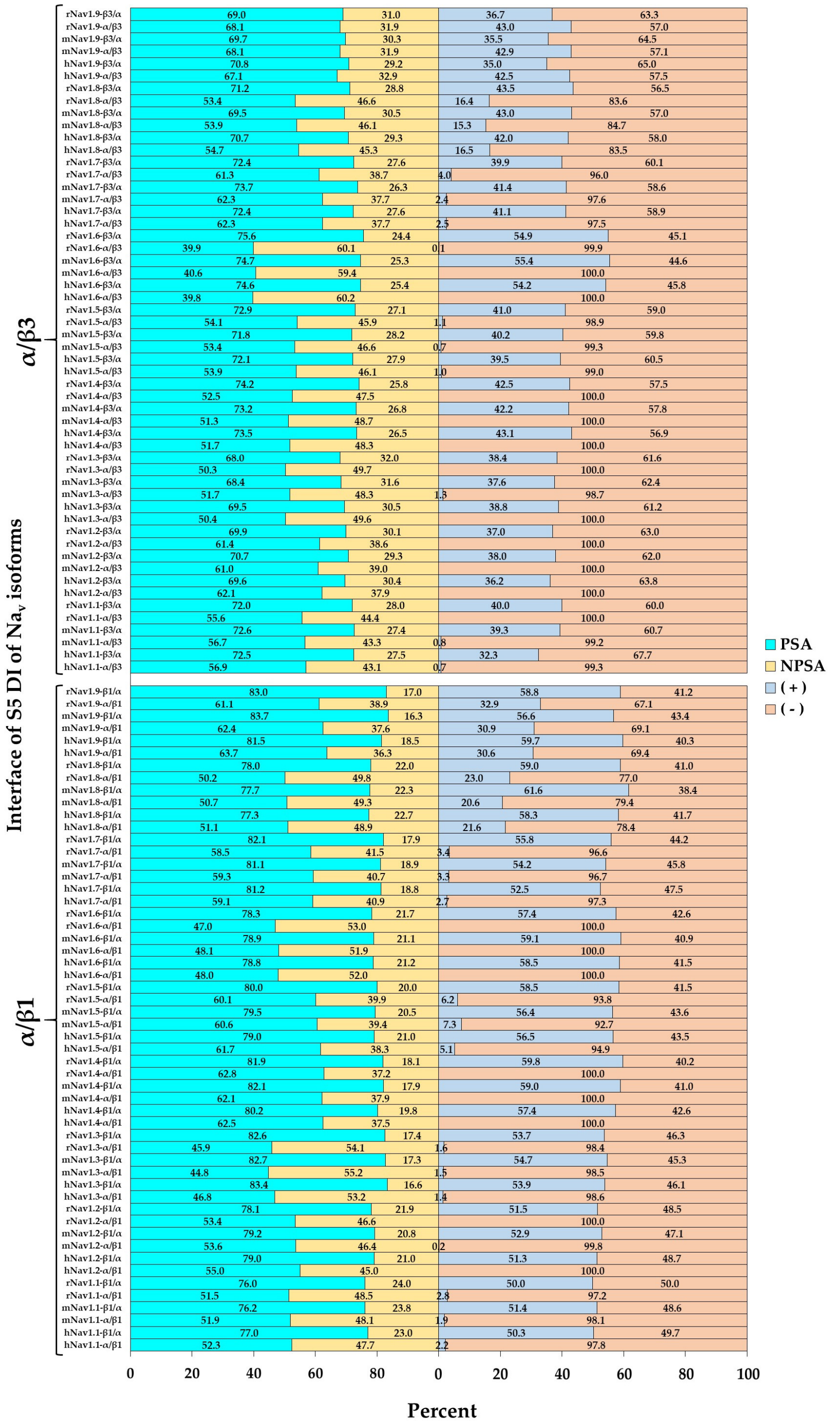

Figure S19. Percentage scores of the surface properties for atoms at Na$_v\alpha$/Na$_v\beta$ of ECL on S5 DI; α/ β: atoms in the α subunit that participate in the interface; β/α: atoms in the β subunit that participate in the interface; PSA: polar surface area; NPSA non-polar surface area; (+): P-MEPS; (-): N-MEPS. The PSA and NPSA represents the total area of the analyzed region.

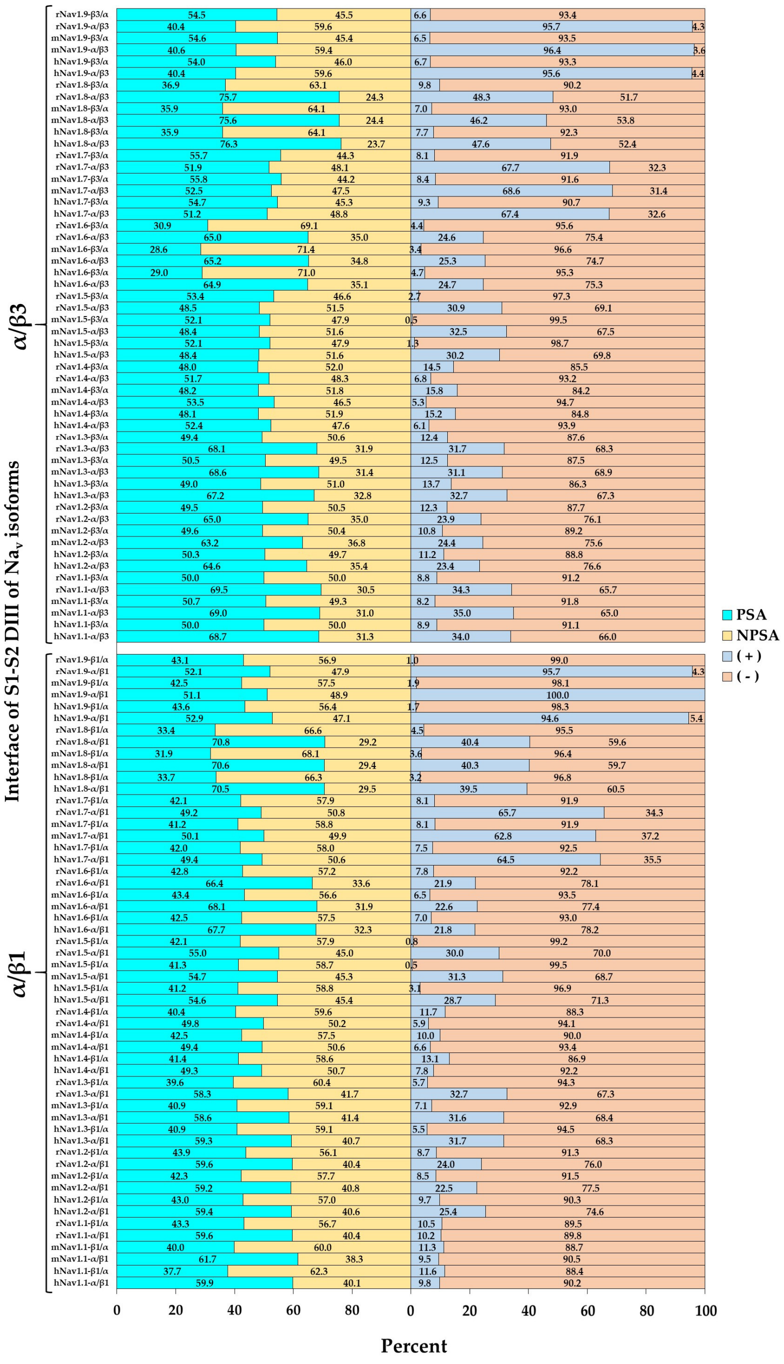

Figure S20. Percentage of the surface properties of atoms that form at the Na$_v$α/Na$_v$β interface of ECL S1-S2 DIII; α/ β: atoms in the α subunit that participate in the interface; β/α: atoms in the β subunit that participate in the interface; PSA: polar surface area; NPSA non-polar surface area; (+): P-MEPS; (-): N-MEPS. The PSA and NPSA represents the total area of the analyzed region.

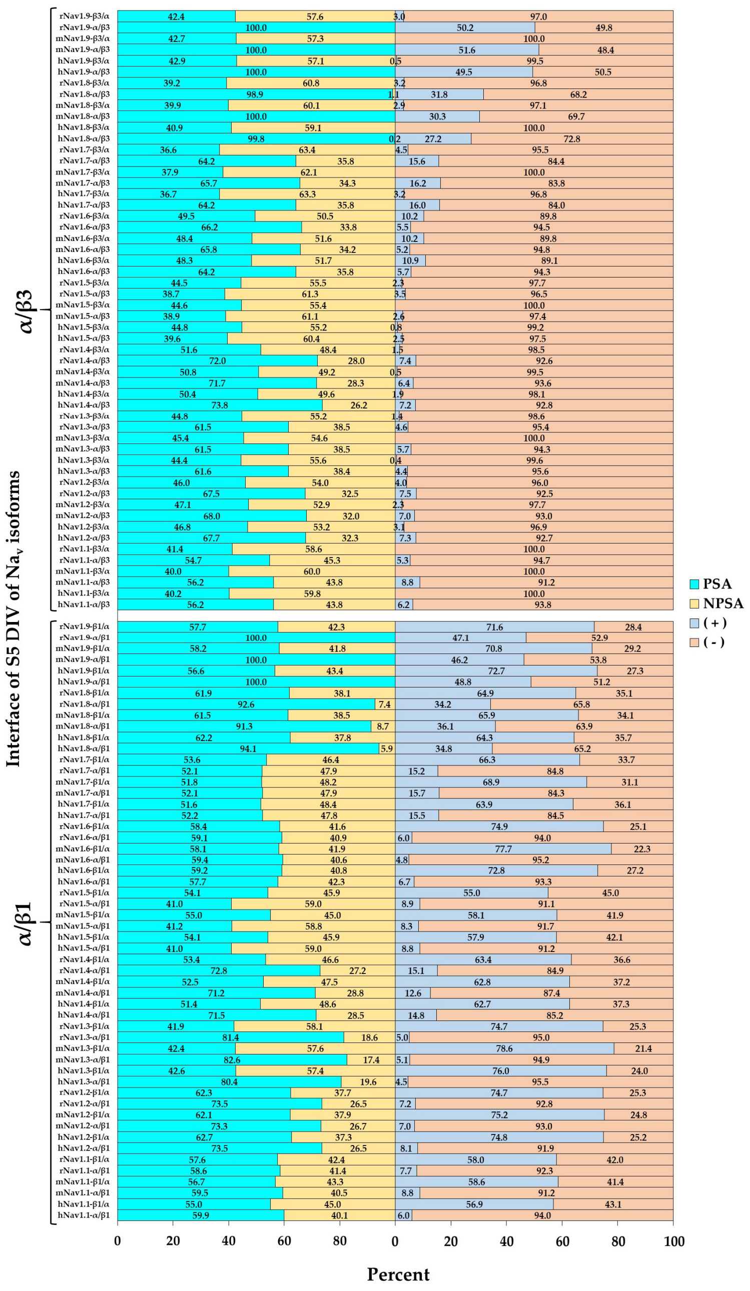

Figure S21. Percentage of the surface properties of atoms that form at the Naᵥα/Naᵥβ interface of ECL S5 DIV; α/ β: atoms in the α subunit that participate in the interface; β/α: atoms in the β subunit that participate in the interface; PSA: polar surface area; NPSA non-polar surface area; (+): P-MEPS; (-): N-MEPS. The PSA and NPSA represents the total area of the analyzed region.

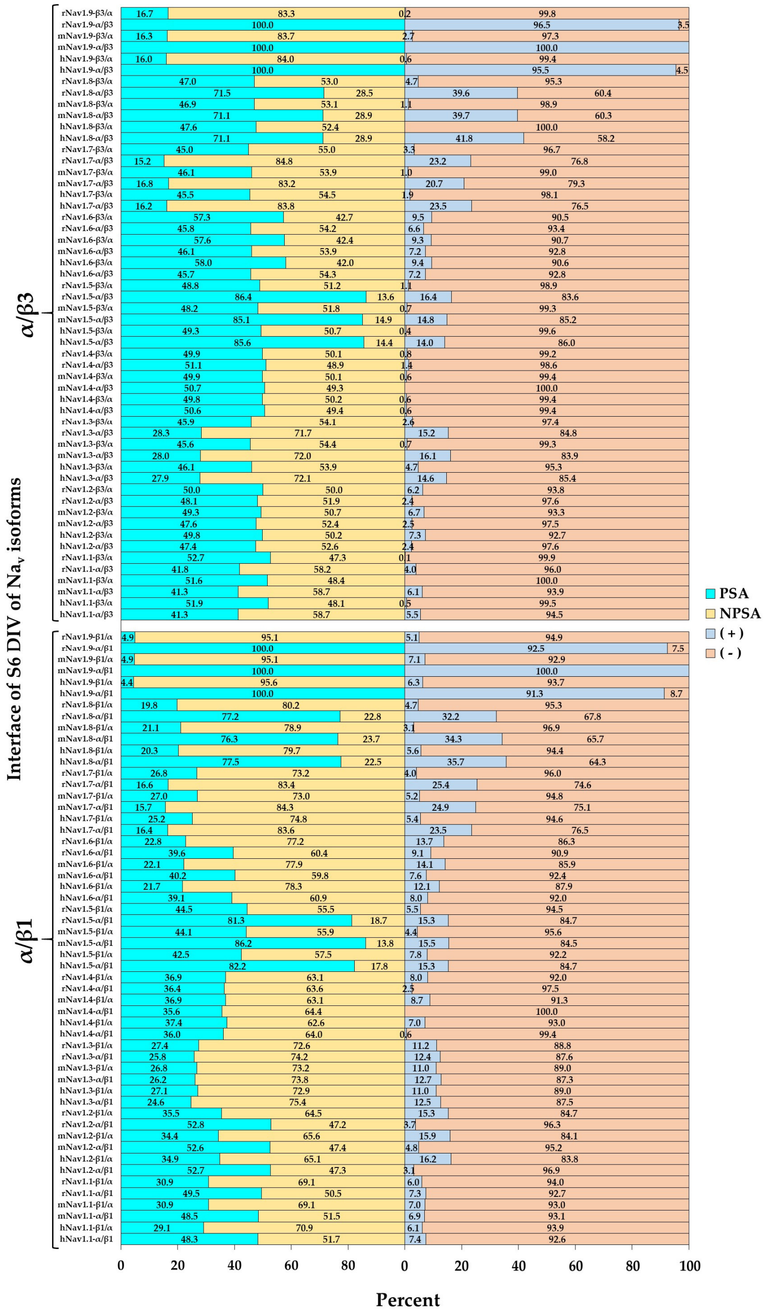

Figure S22. Percentage of the surface properties of atoms that form at the Navα/Navβ interface of ECL S6 DIV; α/ β: atoms in the α subunit that participate in the interface; β/α: atoms in the β subunit that participate in the interface; PSA: polar surface area; NPSA non-polar surface area; (+): P-MEPS; (-): N-MEPS. The PSA and NPSA represents the total area of the analyzed region.

Table S3. Interface area of the IF-ECLs of the Navs

**Nav isoform**	**IF-ECLs of Navs**	**HUMAN**		**MOUSE**		**RAT**	
		**Navβ1**	**Navβ3**	**Navβ1**	**Navβ3**	**Navβ1**	**Navβ3**
Nav1.1	S5 DI	621.8	584.1	664.3	527.2	491.0	367.6
	S1-S2 DIII	294.4	339.8	308.4	299.6	263.0	332.6
	S5 DIV	120.2	151.9	163.1	170.8	137.1	164.6
	S6 DIV	217.5	264.1	270.0	295.9	210.0	327.5
Nav1.2	S5 DI	587.7	552.4	612.8	485.6	535.8	490.1
	S1-S2 DIII	333.8	360.1	350.4	327.3	317.6	380.6
	S5 DIV	136.7	174.5	171.4	158.9	140.5	162.4
	S6 DIV	208.8	254.6	234.0	259.2	220.8	357.5
Nav1.3	S5 DI	602.3	544.5	664.0	554.6	498.0	387.7
	S1-S2 DIII	293.1	330.3	313.2	309.4	315.0	378.9
	S5 DIV	137.9	183.6	171.8	162.6	130.7	135.3
	S6 DIV	187.1	251.5	249.4	272.4	215.4	312.2
Nav1.4	S5 DI	578.6	550.6	559.4	441.0	549.9	434.7
	S1-S2 DIII	304.8	341.4	359.2	350.4	318.0	399.1
	S5 DIV	144.0	170.6	154.6	135.4	129.0	158.5
	S6 DIV	248.2	268.2	284.9	282.3	227.2	300.1
Nav1.5	S5 DI	570.0	534.6	610.2	483.8	535.7	417.3
	S1-S2 DIII	324.7	360.3	368.6	362.0	293.9	357.9
	S5 DIV	175.3	200.0	189.5	184.6	170.9	197.0
	S6 DIV	249.3	281.2	280.7	274.9	218.7	314.3
Nav1.6	S5 DI	525.5	471.6	552.9	440.3	465.3	388.4
	S1-S2 DIII	325.5	360.3	294.8	278.5	287.4	334.5
	S5 DIV	147.2	169.5	167.4	162.1	119.5	145.0
	S6 DIV	199.2	253.7	269.3	288.1	216.1	314.0
Nav1.7	S5 DI	665.5	593.6	663.3	544.9	576.2	461.7
	S1-S2 DIII	381.9	425.8	389.6	380.2	376.9	428.6
	S5 DIV	108.5	141.4	143.9	140.4	89.0	122.7
	S6 DIV	203.5	236.8	232.6	252.1	183.5	261.1
Nav1.8	S5 DI	585.5	551.0	623.7	510.7	583.5	464.8
	S1-S2 DIII	292.5	343.5	279.5	273.7	261.0	330.9
	S5 DIV	179.3	207.6	165.8	136.8	115.7	135.3
	S6 DIV	125.0	162.7	262.0	260.1	211.3	309.4
Nav1.9	S5 DI	633.0	575.7	602.7	520.6	479.9	306.7
	S1-S2 DIII	317.7	385.5	266.2	271.1	298.4	368.2
	S5 DIV	141.6	186.1	138.8	133.1	96.4	128.7
	S6 DIV	17.7	24.9	14.7	25.7	30.0	34.9

The units of area are in Å^2. The interface surface area buried was calculated.

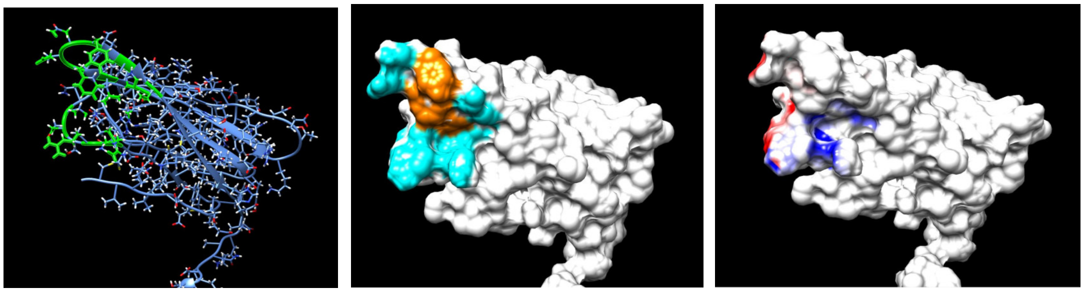

(**a**) (**b**) (**c**)

Figure S23. Demonstration example of the surface of atoms that form the interface in hNa$_v$β1 with the ECL S5 DI in the HMSR of hNa$_v$β1/hNa$_v$1.4 complex. (**a**) atoms that form the interface: green; (**b**) PSA: cyan and NPSA: orange interface area; (**c**) positive P-MEPS interface area: blue and N-MEPS: red; atoms and surface that don't form the interface: cornflower blue and white, respectively. MEPS was calculated for the individual structures and models using the Adaptive Poisson-Boltzmann Solver (APBS) [65] plug-in tool in Chimera Alpha 1.14 [61]. Of note, atom areas that contribute to the interface do not necessarily constitute buried areas (see Table S3).

Validation of the interface residue sequence between Na$_v$α subunit and Na$_v$β1 and Na$_v$β3 subunits

Finally we document the protein-protein interaction zone computed by HOTREGION for the eel template for sodium channel 3D models (PDB code: 5XSY) by a Web-based bioinformatic tool at http://prism.ccbb.ku.edu.tr/hotregion/ . The identified residues correspond to all those which can be studied by Chimera tool, too. For more details consult the software literature [110].

Table S4: The output list of interacting residues between both subunits for the eel sodium channel (PDB code: 5XSY) by HotRegion, a database of cooperative hot spots.

Interface Name	Residue Number	Residue Type	Chain
5XSYBA	273	ARG	A
5XSYBA	301	GLU	A
5XSYBA	313	LEU	A
5XSYBA	323	LYS	A
5XSYBA	324	CYS	A
5XSYBA	325	PRO	A
5XSYBA	326	GLU	A
5XSYBA	327	GLY	A
5XSYBA	985	ILE	A
5XSYBA	988	ASN	A
5XSYBA	989	LEU	A

5XSYBA	992	THR	A
5XSYBA	996	ILE	A
5XSYBA	1005	PHE	A
5XSYBA	1022	ILE	A
5XSYBA	1023	TYR	A
5XSYBA	1025	TRP	A
5XSYBA	1026	ARG	A
5XSYBA	1028	ARG	A
5XSYBA	1032	VAL	A
5XSYBA	1036	TYR	A
5XSYBA	1039	LYS	A
5XSYBA	1040	VAL	A
5XSYBA	1043	TYR	A
5XSYBA	1044	VAL	A
5XSYBA	1047	VAL	A
5XSYBA	1475	TYR	A
5XSYBA	1484	ASP	A
5XSYBA	1489	GLU	A
5XSYBA	1528	ASN	A
5XSYBA	1529	PRO	A
5XSYBA	1530	GLY	A
5XSYBA	1531	THR	A
5XSYBA	24	ALA	B
5XSYBA	25	CYS	B
5XSYBA	26	VAL	B
5XSYBA	27	GLU	B
5XSYBA	28	VAL	B
5XSYBA	31	ASP	B
5XSYBA	45	ILE	B
5XSYBA	47	CYS	B
5XSYBA	49	MET	B
5XSYBA	50	ARG	B
5XSYBA	105	GLN	B
5XSYBA	106	ASP	B
5XSYBA	132	PHE	B

5XSYBA	135	TYR	B
5XSYBA	137	PHE	B
5XSYBA	158	ALA	B
5XSYBA	159	SER	B
5XSYBA	162	SER	B
5XSYBA	166	MET	B
5XSYBA	170	ILE	B
5XSYBA	173	LEU	B
5XSYBA	174	GLN	B
5XSYBA	177	LEU	B
5XSYBA	180	GLU	B
5XSYBA	181	MET	B
5XSYBA	184	CYS	B
5XSYBA	185	TYR	B
5XSYBA	189	ALA	B

The Chimera scripts are documented here which were applied throughout the study.

< Protein – Protein Interface = PPI detection >

measure buriedArea #0.1 #0.2

>Tools>Structure analysis>Attribute calculator

>Insert formula: sum(atom.areaSES)

Select and classif residues

<Superposition of PPIs by Match Maker >

open proteins 1 and 2

commands:

match #2 to #1 bring #3

swapaa (aminoacid name) #1 or #2:Number.(Chain)

mm #2 to #1/a pair bs alg sw matrix PAM-150 ss false cut 5.0

matrix similarity-matrix

The similarity-matrix can be any of: BLOSUM-30, BLOSUM-35, BLOSUM-40, BLOSUM-45, BLOSUM-50, BLOSUM-55, BLOSUM-60, BLOSUM-62 (default), BLOSUM-65, BLOSUM-70, BLOSUM-75, BLOSUM-80, BLOSUM-85, BLOSUM-90, BLOSUM-100, BLOSUM-N, PAM-40, PAM-120, PAM-150, PAM-250, SDM, HSDM, Nucleic.

<Generation of PPI surface, buried surface area and calculate the area>

measure buriedArea #0.1 #0.2

>Tools>Structure analysis>Attribute calculator

>Insert formula: sum(atom.areaSES)

Interf

select @/buriedSESArea> 1

Aplly

< script finish >
